# Kindlin-2 in myoepithelium controls luminal progenitor commitment to alveoli in mouse mammary gland

**DOI:** 10.1038/s41419-023-06184-2

**Published:** 2023-10-13

**Authors:** Zhenbin Wang, Lei Zhang, Bing Li, Jiagui Song, Miao Yu, Jing Zhang, Ceshi Chen, Jun Zhan, Hongquan Zhang

**Affiliations:** 1 Program for Cancer and Cell Biology, Department of Human Anatomy, Histology and Embryology, School of Basic Medical Sciences; Peking University International Cancer Institute; MOE Key Laboratory of Carcinogenesis and Translational Research and State Key Laboratory of Natural and Biomimetic Drugs, Peking University Health Science Center, 100191 Beijing, China; 2 Department of Histology and Embryology, Hebei University of Chinese Medicine, Shijiazhuang, Hebei 050200 China; 3 Academy of Biomedical Engineering, Kunming Medical University, Kunming, 650500 China; 4 Key Laboratory of Animal Models and Human Disease Mechanisms of the Chinese Academy of Sciences and Yunnan Province, Kunming Institute of Zoology, Chinese Academy of Sciences, Kunming, Yunnan 650223 China

**Keywords:** Cell signalling, Differentiation

## Abstract

Myoepithelium plays an important role in mammary gland development, but less is known about the molecular mechanism underlying how myoepithelium controls acinus differentiation during gestation. Herein, we found that loss of Kindlin-2 in myoepithelial cells impaired mammary morphogenesis, alveologenesis, and lactation. Using five genetically modified mouse lines combined with single-cell RNA sequencing, we found a Kindlin-2–Stat3–Dll1 signaling cascade in myoepithelial cells that inactivates Notch signaling in luminal cells and consequently drives luminal progenitor commitment to alveolar cells identity. Single-cell profiling revealed that Kindlin-2 loss significantly reduces the proportion of matured alveolar cells. Mechanistically, Kindlin-2 depletion in myoepithelial cells promotes Stat3 activation and upregulates Dll1, which activates the Notch pathway in luminal cells and inhibits luminal progenitor differentiation and maturation during gestation. Inhibition of Notch1 with tangeretin allowed luminal progenitors to regain commitment ability in the pregnant mice with Kindlin-2 depletion in myoepithelium. Taken together, we demonstrated that Kindlin-2 is essential to myoepithelium-controlled luminal progenitors to alveoli transition during gestation.

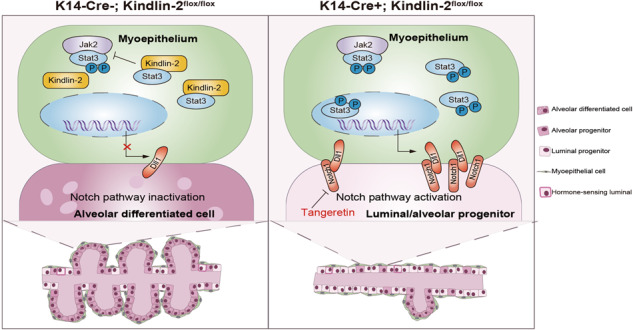

## Introduction

Kindlin-2, encoded by the gene *FERMT2*, is an important integrin-binding protein that is highly expressed in mesoderm-derived organs, including smooth muscle, heart, blood vessel, and bone, but has no or weak expression in endoderm- and ectoderm-derived organs [1]. Global ablation of Kindlin-2 results in peri-implantation lethality at embryonic development day 7.5 (E7.5) in mice [2, 3]. Specific loss of Kindlin-2 impairs smooth muscle formation during embryonic development through the induction of apoptosis. Loss of Kindlin-2 in smooth muscle impairs the contraction of adult smooth muscle by blocking Ca^2+^ influx, which ultimately leads to intestinal obstruction [4]. Kindlin-2 also acts as a critical regulator of myocyte elongation [5] and vascular maintenance and branching [6]. Overexpression of Kindlin-2 in mammary luminal epithelium can promote the growth of gland ductal trees and induce tumor formation [7]. However, the specific role of Kindlin-2 in mammary glands has not been fully elucidated.

Breastfeeding, the normative feeding process for infants, is a public health issue, as it improves the survival of infants and the health of mothers [8, 9]. However, breastfeeding rates are lower than recommended and 5–15% of women suffer from lactation failure worldwide [10]. Development of the mammary gland occurs mainly after birth, including ductal morphogenesis during puberty and acinar morphogenesis during pregnancy [11]. During puberty, morphogenesis is characterized by ductal elongation and branching to form an elaborate ductal tree comprising an inner layer of luminal cells and an outer layer of myoepithelial cells adjacent to the basement membrane. During pregnancy, the mammary epithelium undergoes massive expansion, with the formation of alveolar structures that terminally differentiate into secretory units in late pregnancy to enable lactation. Following weaning, these alveolar structures regress through programmed cell death, returning the gland to a virgin-like state [12].

The Notch signaling pathway is widely reported to participate in mammary stem cell maintenance, cell fate specification, and differentiation during mammary gland development and maturation [13]. A clonal tracing study revealed that Notch1 activation in mammary stem cells is essential for promoting luminal cell specification and driving a progressive transition into unipotent estrogen receptor-negative luminal progenitors [14]. During the final trimester of pregnancy and lactation, Notch pathway activation appears to be reduced, including both the distributions of receptors and ligands in mammary epithelial cells and expression of target genes [15, 16]. These findings demonstrate the dynamic regulation of the Notch pathway in luminal cells, but the mechanisms through which these ligands of the Notch pathway are regulated require further investigation.

In this study, using single-cell profiling in combination with multiple genetically modified mouse models, we demonstrate that Kindlin-2 is an essential regulatory element in the myoepithelium that maintains a suitable maturation niche for the luminal epithelium of the mammary gland. We describe a novel mechanism through which Kindlin-2 plays an indispensable role in the fate determination of luminal and alveolar progenitors in myoepithelial cells via regulation of the Jak2/Stat3/Dll1 axis and Notch pathway in alveolar cells.

## Results

### Mammary epithelial-specific knockout of Kindlin-2 inhibits lactation in female mice

**Fig. 1:**
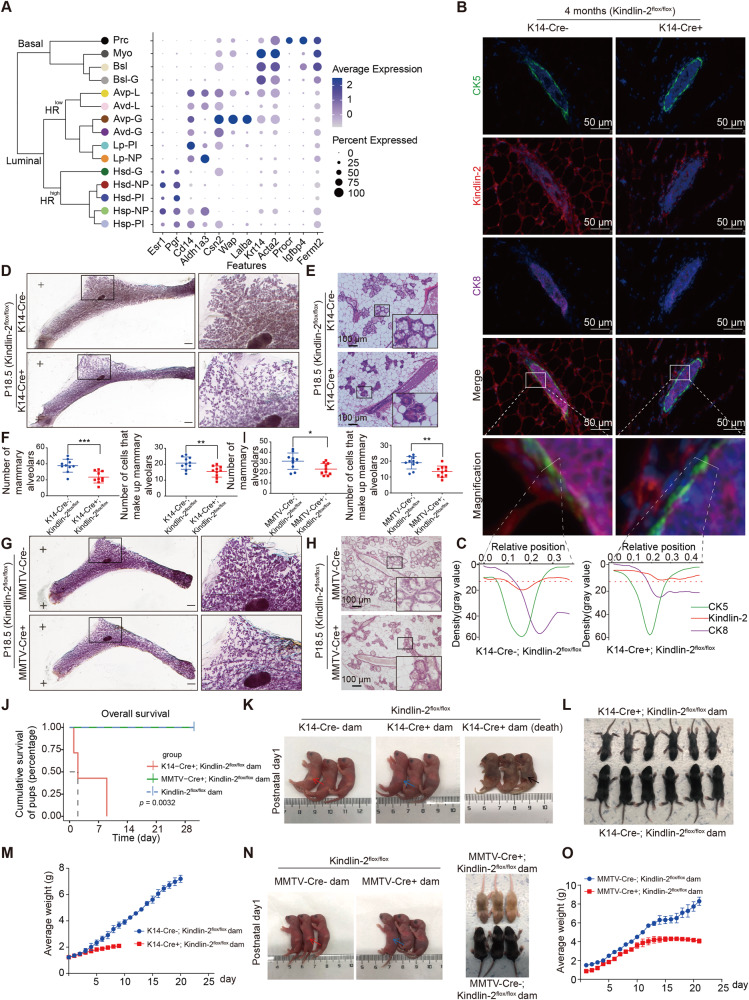
Mammary epithelial-specific knockout of Kindlin-2 inhibits lactation of female mice. **A** Dot plot representing the expression level (purple jet) and the number of expressing cells (dot size) of canonical markers from each cell lineages and *FERMT2* in mammary epithelial cells (GSE106273). **B** Multi-color staining showing CK5 (green), Kindlin-2 (red), CK8 (purple), and DAPI (blue) in 4-month-old mammary gland of K14-Cre + ; Kindlin-2^flox/flox^ or K14-Cre-; Kindlin-2^flox/flox^ littermate control mice. **C** Quantitative analysis the relative positions of CK5, Kindlin-2, and CK8 fluorescence intensities in 4-month-old mammary gland of K14-Cre + ; Kindlin-2^flox/flox^ or K14-Cre-; Kindlin-2^flox/flox^ littermate control mice by ImageJ. **D** Whole-mounted staining of P18.5 mammary gland in K14-Cre + ; Kindlin-2^flox/flox^ or K14-Cre-; Kindlin-2^flox/flox^ littermate control mice. **E** HE sections staining of P18.5 mammary gland in K14-Cre + ; Kindlin-2^flox/flox^ or K14-Cre-; Kindlin-2^flox/flox^ littermate control mice. Scale bars, 100 µm. **F** Statistical analysis showing the number of mammary alveolars and cells that make up mammary alveolars in K14-Cre + ; Kindlin-2^flox/flox^ group compared with K14-Cre-; Kindlin-2^flox/flox^ group (*n* = 10 fields of view at random per genotype). Statistical testing was performed by unpaired *t* test. Data are presented as mean values ± SD. *P < 0.05, **P < 0.01, ***P < 0.001. **G** Whole-mounted staining of P18.5 mammary gland in MMTV-Cre + ; Kindlin-2^flox/flox^ or MMTV-Cre-; Kindlin-2^flox/flox^ littermate control mice. **H** HE sections staining of P18.5 mammary gland in MMTV-Cre + ; Kindlin-2^flox/flox^ or MMTV-Cre-; Kindlin-2^flox/flox^ littermate control mice. Scale bars, 100 µm. **I** Statistical analysis showing the number of mammary alveolars and cells that make up mammary alveolars in MMTV-Cre+; Kindlin-2^flox/flox^ group compared with MMTV-Cre-; Kindlin-2^flox/flox^ group (*n* = 10 fields of view at random per genotype). Statistical testing was performed by unpaired *t* test. Data are presented as mean values ± SD. *P < 0.05, **P < 0.01, ***P < 0.001. **J** Postpartum pups survival percent change in MMTV-Cre + ; Kindlin-2^flox/flox^ (*n* = 3), K14-Cre + ; Kindlin-2^flox/flox^ (*n* = 7), and Kindlin-2^flox/flox^ group (*n* = 3). Statistical testing was performed by Log-Rank test. **K** Comparing mice one day after birth in K14-Cre + ; Kindlin-2^flox/flox^ group with K14-Cre-; Kindlin-2^flox/flox^ group. Arrows indicate the stomach of the mice. **L** Reduced growth of pups nursed by lactating K14-Cre + ; Kindlin-2^flox/flox^ dams compared with control littermate females. Representative photographs of pups are shown in (**L**). **M** Comparing postpartum pups weights change curve in K14-Cre + ; Kindlin-2^flox/flox^ group (*n* = 3) with K14-Cre-; Kindlin-2^flox/flox^ group (*n* = 3). Blue represents K14-Cre-; Kindlin-2^flox/flox^ group. Red represents K14-Cre + ; Kindlin-2^flox/flox^ group. Data are presented as mean values ± SD. **N** Left: Comparing dam mice one day after birth in MMTV-Cre + ; Kindlin-2^flox/flox^ group with MMTV-Cre-; Kindlin-2^flox/flox^ group. Arrows indicate the stomach of the mouse. Right: Growth of pups nursed by lactating MMTV-Cre + ; Kindlin-2^flox/flox^ dams compared with control littermate females. Representative photographs of pups are shown. **O** Comparing postpartum pups weights change curve in MMTV-Cre + ; Kindlin-2^flox/flox^ group (*n* = 3) with MMTV-Cre-; Kindlin-2^flox/flox^ group (*n* = 3). Blue represents MMTV-Cre-; Kindlin-2^flox/flox^ group. Red represents MMTV-Cre + ; Kindlin-2^flox/flox^ group. Data are presented as mean values ± SD.

We first acquired a report of the single-cell landscape of the mammary gland during puberty, gestation, lactation, and the involution stage to determine the physiological distribution of Kindlin-2 in mammary glands [17]. Interestingly, Kindlin-2 was mainly expressed in basal and myoepithelial cells, consistent with the important function of Kindlin-2 in smooth muscle tissues and basal-like breast cancer (Fig. 1A).

To fully explore the function of Kindlin-2 in the mammary gland, we generated myoepithelial cell-specific (K14-Cre) and mammary luminal cell-specific (MMTV-Cre) Kindlin-2 gene knockout mice using the Cre-LoxP system (Supplementary Fig. 1A). The K14-Cre + ; Kindlin-2^flox/flox^ genotype was distinguished through a polymerase chain reaction (PCR) assay (Supplementary Fig. 1B). Western blotting showed that the level of Kindlin-2 was reduced in breast tissues but not kidney or liver tissues (Supplementary Fig. 1C). Immunohistochemistry demonstrated a reduction of Kindlin-2 in the basal layer of mammary ducts in the K14-Cre + ; Kindlin-2^flox/flox^ group (Supplementary Fig. 1D). Due to the difficulty of distinguishing myoepithelial cells in the immunohistochemistry assay, we applied multi-color immunofluorescence staining to explore the localization and expression of Kindlin-2 in myoepithelial cells of wild-type and K14-Cre + ; Kindlin-2^flox/flox^ adult mice (Fig. 1B). Consistent with the previously reported single-cell landscape, Kindlin-2 showed higher expression levels in myoepithelial cells, which were marked with cytokeratin 5 (CK5), of the Kindlin-2^flox/flox^ group and was apparently knocked out in the K14-Cre + ; Kindlin-2^flox/flox^ group (Fig. 1C). The genotype of MMTV-Cre could be distinguished based on mouse coat color, with a yellow coat representing the MMTV-Cre + ; Kindlin-2^flox/flox^ genotype and a black coat representing the Kindlin-2^flox/flox^ genotype (Supplementary Fig. 1E). In addition, the genotype was characterized using PCR (Supplementary Fig. 1F). To confirm the specificity and efficiency of MMTV promoter-based Cre expression, we detected the levels of Kindlin-2 in the lung, breast, and kidney. Western blotting showed that the level of Kindlin-2 was significantly down-regulated in breast tissue but had no change in the other tissues tested (Supplementary Fig. 1G). Immunohistochemistry results supported the down-regulation of Kindlin-2 in mammary luminal cells of the MMTV-Cre + ; Kindlin-2^flox/flox^ group (Supplementary Fig. 1H). These results demonstrated that Kindlin-2 knockout mouse models for mammary myoepithelial or luminal cells were successfully generated.

Furthermore, we monitored the morphology of the mammary gland in K14-Cre + ; Kindlin-2^flox/flox^, MMTV-Cre + ; Kindlin-2^flox/flox^, and Kindlin-2^flox/flox^ female virgin mice for 4 months, and whole-mount staining with carmine showed that the number of tertiary branches was significantly reduced in both the K14-Cre + ; Kindlin-2^flox/flox^ group and MMTV-Cre + ; Kindlin-2^flox/flox^ group compared with the Kindlin-2^flox/flox^ group (Supplementary Fig. 1I, J). These results suggest that depletion of Kindlin-2 impairs morphogenesis of the mammary gland.

Analysis of mammary glands during pregnancy in either K14-Cre + ; Kindlin-2^flox/flox^ or MMTV-Cre + ; Kindlin-2^flox/flox^ females indicated an important role of Kindlin-2 in alveolar expansion. The formation of alveolar units was significantly compromised in Kindlin-2-deficient females at 18.5 days of pregnancy in the K14-Cre + ; Kindlin-2^flox/flox^ group, as reflected by the presence of small, sparsely distributed alveoli (Fig. 1D, E). The number of mammary alveoli and the number of cells that make up mammary alveoli were significantly reduced in K14-Cre + ; Kindlin-2^flox/flox^ mice (Fig. 1F). Similar to the K14-Cre + ; Kindlin-2^flox/flox^ group, the formation of alveolar units was significantly compromised in Kindlin-2-deficient females at 18.5 days of pregnancy in the MMTV-Cre + ; Kindlin-2^flox/flox^ group (Fig. 1G–I). These results indicate that loss of Kindlin-2 affects alveologenesis in the mouse mammary gland during gestation.

The ability to feed pups is one of the most important indicators for evaluating mammary function. We found that all pups produced by K14-Cre + ; Kindlin-2^flox/flox^ female mice died within 9 days, whereas pups produced by Kindlin-2^flox/flox^ and MMTV-Cre + ; Kindlin-2^flox/flox^ survived (Fig. 1J). These findings indicated that K14-Cre + ; Kindlin-2^flox/flox^ dams were completely incapable of nursing their pups, whereas MMTV-Cre + ; Kindlin-2^flox/flox^ and Kindlin-2^flox/flox^ dams could feed their pups until ablactation.

Examination of K14-Cre + ; Kindlin-2^flox/flox^ and Kindlin-2^flox/flox^ dams revealed that pups nursed by K14-Cre + ; Kindlin-2^flox/flox^ dams were significantly stunted, with little milk present in their stomachs (Fig. 1K). Severely affected mice began dying on the day of birth and attempting to supplement the dams with nutrition did not improve the condition of their pups (Fig. 1K). We found that the mice in the experimental group of the same age were significantly smaller than mice in the control group at 8 days after birth (Fig. 1L). Average weight was significantly reduced in pups nursed by K14-Cre + ; Kindlin-2^flox/flox^ dams compared to Kindlin-2^flox/flox^ dams at the early stage of lactation (Fig. 1M). These results suggest that loss of Kindlin-2 in myoepithelial cells catastrophically impairs lactation.

Examination of MMTV-Cre + ; Kindlin-2^flox/flox^ and Kindlin-2^flox/flox^ dams revealed that pups nursed by MMTV-Cre + ; Kindlin-2^flox/flox^ dams were relatively stunted, with a little milk present in their stomachs (Fig. 1N). Average weight was significantly lower in pups nursed by MMTV-Cre + ; Kindlin-2^flox/flox^ dams compared to Kindlin-2^flox/flox^ dams, especially after 10 days of nursing (Fig. 1O). To exclude effects of pup genotype, pups of MMTV-Cre + ; Kindlin-2^flox/flox^ were nursed by Kindlin-2^flox/flox^ dams beginning at 14 days after parturition, and the results showed that pups nursed by Kindlin-2^flox/flox^ dams regained normal weight (Supplementary Fig. 1K). In contrast with pups fed by K14-Cre + ; Kindlin-2^flox/flox^ dams, none of the pups fed by MMTV-Cre + ; Kindlin-2^flox/flox^ dams starved to death, with most instead showing malnutrition relative to the Kindlin-2^flox/flox^ group.

Based on the distribution of Kindlin-2 and the difference in nursing ability among epithelium-specific knockout mouse models, we proposed that Kindlin-2 in the myoepithelium plays an essential role in controlling the development and lactation function of the mammary gland.

### Single-cell RNA-seq reveals the impact of Kindlin-2 depletion on the mammary gland microenvironment during the final trimester of pregnancy

**Fig. 2:**
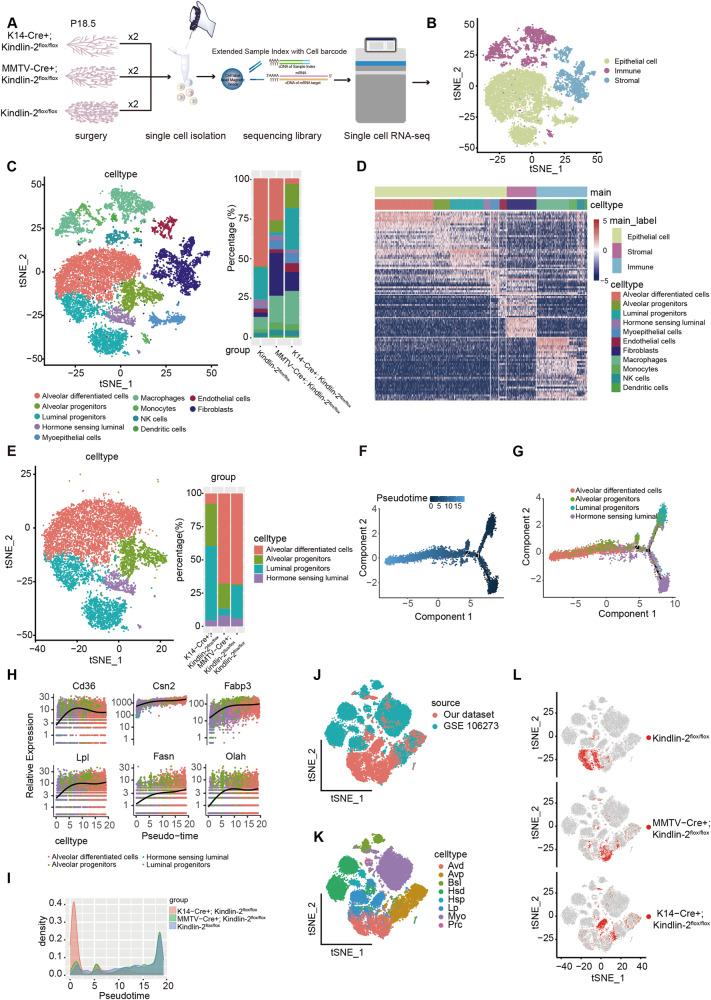
Single cell RNAs-seq reveals the impactions of loss of Kindlin-2 in mammary gland microenvironment during late trimester of pregnancy. **A** Workflow showing collection and processing of Kindlin-2^flox/flox^, K14-Cre + ; Kindlin-2^flox/flox^ and MMTV-Cre + ; Kindlin-2^flox/flox^ in P18.5 mouse mammary gland samples for generating scRNA-seq data. **B** T-SNE plot showing of all 15719 classified cells, demonstrating separation of cells by main cell lineages. **C** T-SNE plot of the distribution of the cell lineage and their relative percentage (right). **D** Heatmap showing distinct features of each cell lineages. Rows, genes. Columns, cells. The color key from blue to red indicates low to high gene expression. **E** T-SNE plot of the distribution of the luminal cells lineages and their relative percentage. **F**, **G** Developmental trajectory of luminal cell lineages constructed by Monocle2 and colored by cells lineages and pseudotime. **H** Expression levels of indicated lactation related genes with respect to their pseudotime coordinates. Black lines depict the LOESS regression fit of the normalized expression values. **I** Density plot of the luminal cell lineages from each genotype along with the pseudotime coordinates. **J** T-SNE plot showing the distribution of the dataset source. **K** T-SNE plot showing the distribution of cell lineages within the integrated dataset combining our different genotype P18.5 mammary glands and physiological different states mammary glands (GSE106273). **L** T-SNE plot showing the distribution of different genotype mammary epithelial cells in the integrated dataset.

To reveal the mechanism through which knockout of Kindlin-2 in myoepithelial cells induced disorders during gestation and lactation, we isolated a total of six mammary glands from K14-Cre + ; Kindlin-2^flox/flox^, MMTV-Cre + ; Kindlin-2^flox/flox^, and Kindlin-2^flox/flox^ mice at 18.5 days of gestation for single-cell RNA-seq analysis (Fig. 2A). After low-quality cells were filtered out through Seurat analysis, 15,719 high-quality single-cell transcriptomes were retained and profiled, and an average of 2120 genes and 11,385 counts per sample were detected (Supplementary Fig. 2A) [18]. We reduced dimensionality through principal component analysis and visualized the data using t-distributed stochastic neighbor embedding. Cells with similar expression patterns were clustered using a graph-based clustering method (Supplementary Fig. 2B). Using marker genes for major cell lineages, cells were annotated as epithelial cells, immune cells, and stromal cells (Fig. 2B and Supplementary Fig. 2C).

Based on differential gene expression analysis and known cell type references, cells were divided into four immune compartments (T/NK cells, dendritic cells, macrophages, and monocytes), a stromal compartment (fibroblasts), and six epithelial compartments (alveolar differentiated cells, alveolar progenitors, luminal progenitors, hormone-sensing luminal cells, myoepithelial cells, and endothelial cells). We found that the mammary glands of different groups showed distinct distributions of cell lineages (Fig. 2C). The most highly expressed genes are shown in Supplementary Fig. 2D, E. Aside from myoepithelial cells, mammary epithelial cells displayed a similar expression pattern of a gradual shift from immature luminal progenitors to alveolar differentiated cells fully differentiated for lactation (Fig. 2D). Based on the distribution of cell lineages, we determined that K14-Cre + ; Kindlin-2^flox/flox^ mammary glands contained few mature alveolar cells and fewer progenitor cells compared to other groups. This disproportionate cell type distribution was improved in the MMTV-Cre + ; Kindlin-2^flox/flox^ mammary gland. This change in the cell distribution is in accordance with our histological observations and estimates of feeding capacity among the three groups.

To explore the function of Kindlin-2 in mammary epithelial maturation and differentiation in detail, we collected mammary luminal epithelial cells and took a census of cell lineages (Fig. 2E). We also inspected the expression levels of Kindlin-2 in myoepithelial cells and luminal cells. Kindlin-2 was accurately knocked out in the K14-Cre + ; Kindlin-2^flox/flox^ myoepithelial cells, while the MMTV-Cre + ; Kindlin-2^flox/flox^ group showed no significant difference in luminal epithelium compared with other groups due to the relatively low Kindlin-2 expression levels typical of luminal cells (Supplementary Fig. 3A).

Monocle 2 was used to construct a pseudotime map [19–21], which revealed the trajectory of the luminal cell maturation process (Fig. 2F). Mammary luminal epithelial cells were divided into five states with two branch points (Supplementary Fig. 3B). Cells from luminal progenitors were mainly distributed in state 4, one of the initial branches along the pseudotime trajectory, while hormone-sensing luminal cells settled in state 3, the other early branch. Alveolar differentiated cells were located in state 1, at the terminal of the pseudotime trajectory, and alveolar progenitors were continuously situated along the branch from luminal progenitors to alveolar differentiated cells (Fig. 2G and Supplementary Fig. 3C). The trajectory we constructed was consistent with the reported physiological differentiation trajectory. We also observed that the expression levels of milk-related genes (*Cd36*, *Csn2*, *Fabp3*, *Fasn*, *Lpl*, and *Olah*) increased gradually with maturation and terminal differentiation (Fig. 2H) [22]. Mapping group information to the trajectory, we observed that luminal cells in the K14-Cre + ; Kindlin-2^flox/flox^ group were mainly located along the two early branches, while cells from MMTV-Cre + ; Kindlin-2^flox/flox^ and Kindlin-2^flox/flox^ dominated the terminal development trajectory (Fig. 2I and Supplementary Fig. 3D).

To verify whether our luminal cell differentiation trajectory was in accordance with previous research, we integrated our dataset with reports of mammary epithelial cells, covering mammary glands at the virgin, pregnancy, lactation, and involution stages (Fig. 2J) [17]. The integrated results covered the processes of luminal cell differentiation, maturation, and involution throughout the reproductive cycle (Fig. 2K). Our dataset complements the terminal differentiation of luminal cells during late gestation (Supplementary Fig. 3E). Combined with the data presented above, the findings indicate that loss of Kindlin-2 in myoepithelial cells impedes the terminal differentiation and maturation of luminal progenitors (Fig. 2L).

### Myoepithelium-specific knockout of Kindlin-2 activates the Notch pathway in the luminal epithelium

Mammary gland development and lactation require complex and dynamic stimulation from intercellular signals, intracellular regulatory processes, and the extracellular matrix. Intercellular communication is important for maintaining the balance of the mammary gland microenvironment and disequilibrium of intercellular communication might induce dysfunction. We used CellChat to investigate intercellular communication disparities between K14-Cre + ; Kindlin-2^flox/flox^ and Kindlin-2^flox/flox^ mouse mammary epithelial cells (Supplementary Fig. 4A, B) [23]. Some signaling pathways, including Notch, Fgf, and Ptn, were generally enriched in the K14-Cre + ; Kindlin-2^flox/flox^ group, while other pathways such as Lifr and Tgf-beta were mainly enriched in the Kindlin-2^flox/flox^ group (Supplementary Fig. 4C).

**Fig. 3:**
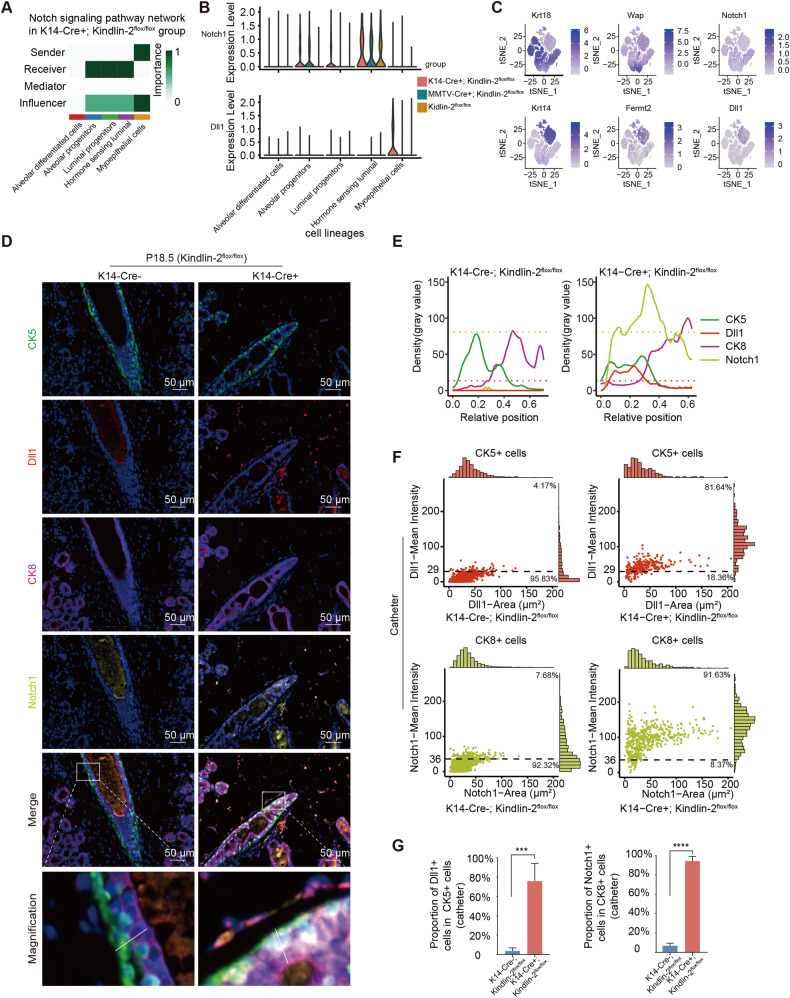
Myoepithelial cells specific knockout of Kindlin-2 activates Notch signaling pathway in P18.5 mammary gland by upregulating Dll1 in myoepithelium and Notch1 in luminal epithelium. **A** Heatmap of Notch pathway interactions network within K14-Cre + ; Kindlin-2^flox/flox^ genotype mammary epithelial cells. **B** Violin plots showing Notch1, Dll1, *FERMT2* expression levels among mammary epithelial cells of each genotype. **C** T-SNE plot showing the expression levels of luminal cell markers (*Krt18*, *Wap*), Notch receptor (*Notch1*), myoepithelial cell markers (*Krt4*, *Fermt2*) and Notch ligand (*Dll1*) in integrated data. The color key from gray to purple indicates low to high gene expression. **D** Multi-color staining showing CK5 (green), Dll1 (red), CK8 (purple), Notch1 (yellow), and DAPI (blue) of P18.5 mammary gland in K14-Cre + ; Kindlin-2^flox/flox^ or K14-Cre-; Kindlin-2^flox/flox^ littermate control mice. **E** Quantitative analysis the relative positions of CK5, Dll1, CK8, and Notch1 fluorescence intensities of P18.5 mammary gland in K14-Cre + ; Kindlin-2^flox/flox^ or K14-Cre-; Kindlin-2^flox/flox^ littermate control mice by ImageJ. **F** The scatter plot showing the number of Dll1+ cells in CK5+ cells, and Notch1+ cells in CK8+ cells of P18.5 mammary gland catheters in K14-Cre + ; Kindlin-2^flox/flox^ or K14-Cre-; Kindlin-2^flox/flox^ females. **G** Statistical analysis showing the proportion of Dll1+ cells in CK5+ cells, and Notch1+ cells in CK8+ cells at P18.5 in K14-Cre + ; Kindlin-2^flox/flox^ group compared with K14-Cre-; Kindlin-2^flox/flox^ group (*n* = 6 catheters of view at random per genotype). Statistical testing was performed by unpaired *t* test. Data are presented as mean values ± SD. *P < 0.05, **P < 0.01, ***P < 0.001, ****P < 0.0001.

Knockout of Kindlin-2 in myoepithelial cells strikingly increased activity of the Notch pathway, a canonical pathway mediating the proliferation and stemness of mammary luminal cells, as a result of the elevation of Dll1, a classical Notch pathway ligand (Fig. 3A, B and Supplementary Fig. 4D-F) [13, 24, 25]. The major senders of the Notch signal were myoepithelial cells and the receivers included all types of immature luminal cells (Fig. 3B). We checked other pairs of ligands and receptors associated with the Notch pathway to verify that Dll1 and Notch1 were the only ligand and receptor regulated in our experiment (Supplementary Fig. 4D). We also estimated the expression of Dll1 and Notch1 in the mammary gland throughout the reproductive cycle and found that Dll1 was mainly expressed in the basal and myoepithelial cells, while Notch1 was primarily expressed in immature progenitors, which exhibited lower levels of canonical lactation-related markers (Fig. 3C), echoing previous research [16].

To verify the alterations in the Notch pathway induced by loss of Kindlin-2 in myoepithelial cells, we employed multi-color immunofluorescence staining to compare the localization and expression of Notch1 and Dll1 between myoepithelial cells, marked with CK5, and luminal cells, marked with CK8, in 18.5-day pregnant Kindlin-2^flox/flox^ and K14-Cre + ; Kindlin-2^flox/flox^ mice (Fig. 3D). Protein levels of Dll1 were significantly upregulated in the myoepithelium of the K14-Cre + ; Kindlin-2^flox/flox^ group, and Notch1 was more highly expressed in luminal cells compared with the Kindlin-2^flox/flox^ group (Fig. 3E–G). Similar results were obtained in mammary alveoli (Supplementary Fig. 5A, B). Using immunohistochemistry assays, we also found that the target gene of the Notch pathway, Hes1, was elevated in luminal cells of K14-Cre + ; Kindlin-2^flox/flox^ mice (Supplementary Fig. 4G).

Consistent with the single-cell RNA-seq results, we confirmed that the mammary gland of the K14-Cre + ; Kindlin-2^flox/flox^ group exhibited strong Notch pathway communication and activation during the final trimester of pregnancy, with high expression levels of the ligand in myoepithelial cells and luminal cells remaining in a poorly differentiated state with high levels of Notch1 expression.

### Myoepithelium-specific Kindlin-2 knock-in inhibits Notch pathway activation in the luminal epithelium

**Fig. 4:**
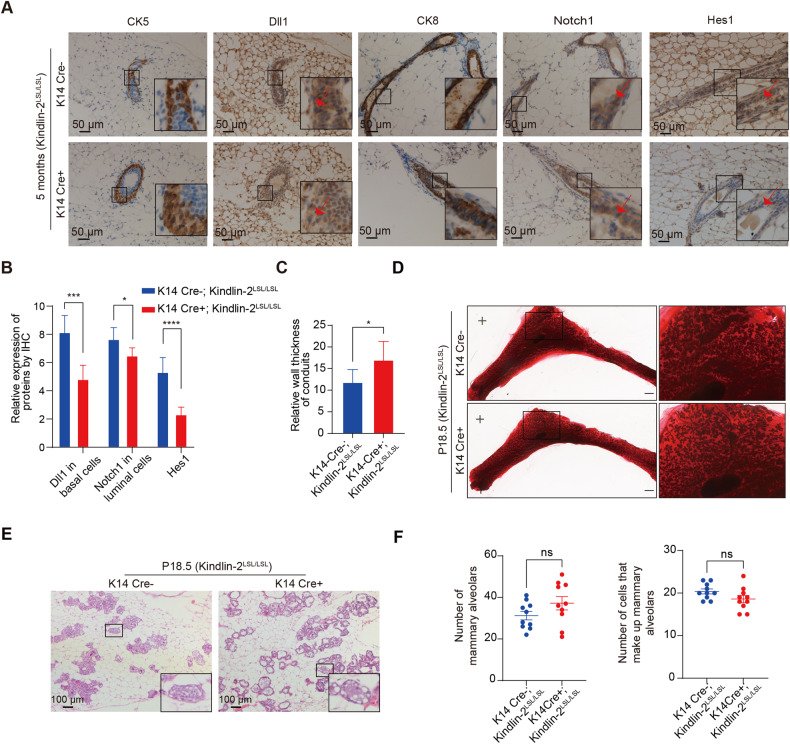
Myoepithelium specific Kindlin-2 knock-in inhibits the activation of the Notch signaling pathway in mammary glandular ducts. **A** Evaluation of Dll1 levels in basal cells (CK5 positive), Notch1 levels in luminal cells (CK8 positive) and the target protein of notch signaling pathway Hes1 levels in 5-month-old mammary gland ducts of K14-Cre + ; Kindlin-2^LSL/LSL^ compared with K14-Cre-; Kindlin-2^LSL/LSL^ littermate control mice by IHC with indicated antibodies. In order to better locate myoepithelial cells and luminal epithelial cells, our staining was performed on adjacent slices. Scale bar, 50 µm. **B** Quantification for the Dll1 levels in basal cells (CK5 positive), Notch1 levels in luminal cells (CK8 positive) and the target protein of Notch signaling pathway Hes1 levels in the ducts of K14-Cre + ; Kindlin-2^LSL/LSL^ mammary gland compared with K14-Cre-; Kindlin-2^LSL/LSL^ mammary gland (*n* = 6). Statistical testing was performed by unpaired *t* test. Data are presented as mean values ± SD. *P *<* 0.05, **P < 0.01, ***P < 0.001, ****P < 0.0001. **C** Quantification for the thickness of mammary gland ducts at the age of 5 months from K14-Cre + ; Kindlin-2^LSL/LSL^ mammary gland compared with K14-Cre-; Kindlin-2^LSL/LSL^ mammary gland (*n* = 6). Statistical testing was performed by unpaired *t* test. Data are presented as mean values ± SD. *P < 0.05. **D** Whole-mounted staining of P18.5 mammary gland in K14-Cre + ; Kindlin-2^LSL/LSL^ or K14-Cre-; Kindlin-2^LSL/LSL^ littermate control mice. **E** HE sections staining of P18.5 mammary gland in K14-Cre + ; Kindlin-2^LSL/LSL^ or K14-Cre-; Kindlin-2^LSL/LSL^ littermate control mice. Scale bars, 100 µm. **F** Statistical analysis showing the number of mammary alveolars and cells that make up mammary alveolars in K14-Cre + ; Kindlin-2^LSL/LSL^ group compared with K14-Cre-; Kindlin-2^LSL/LSL^ group (*n* = 10 fields of view at random per genotype). Statistical testing was performed by unpaired *t* test. Data are presented as mean values ± SEM. *P < 0.05, **P < 0.01, ***P < 0.001.

Based on our initial results, we observed that depletion of Kindlin-2 in myoepithelial cells significantly impacts mammary gland development and maturation. To clarify whether overexpression of Kindlin-2 in myoepithelial cells boosts mammary gland development, we constructed K14 Cre + ; Kindlin-2^LSL/LSL^ mice, which expressed higher levels of Kindlin-2 in myoepithelial cells than Kindlin-2^LSL/LSL^ (Supplementary Fig. 6A–C). We detected mammary gland morphology in K14 Cre + ; Kindlin-2^LSL/LSL^ and Kindlin-2^LSL/LSL^ female virgin mice at 5 months of age, and whole-mount carmine staining showed that the number of tertiary branches was greater in the K14 Cre + ; Kindlin-2^LSL/LSL^ group than the Kindlin-2^LSL/LSL^ group (Supplementary Fig. 6D, E). We calculated the average intensity of Dll1, Notch1 and Hes1 expression in certain unit cells and found that the expression levels of Dll1 in myoepithelial cells and of Notch1 and Hes1 in luminal epithelial cells were apparently reduced in mammary glands of the K14 Cre + ; Kindlin-2^LSL/LSL^ group (Fig. 4A, B). Meanwhile, the relative wall thickness of conduits was greater in the K14 Cre + ; Kindlin-2^LSL/LSL^ group (Fig. 4C). We obtained insights into the development of mammary glands during pregnancy, and demonstrated that the number of mammary alveoli and the number of cells that make up mammary alveoli were similar in the K14 Cre + ; Kindlin-2^LSL/LSL^ group and the Kindlin-2^LSL/LSL^ group; this result revealed that overloading of Kindlin-2 in the myoepithelium did not significantly promote alveologenesis. These findings indicate that overexpression of Kindlin-2 inhibits Dll1/Notch1 cross-talk between myoepithelial cells and luminal cells, thereby enhancing mammary duct development. However, in maturation, the intrinsic mechanism of Kindlin-2 is to safeguard the development of the mammary gland during gestation. The formation of alveoli showed no distinct difference between the K14 Cre + ; Kindlin-2^LSL/LSL^ group and the K14 Cre-; Kindlin-2^LSL/LSL^ group (Fig. 4D–F).

### Kindlin-2 interacts with and inhibits the activation of Stat3

**Fig. 5:**
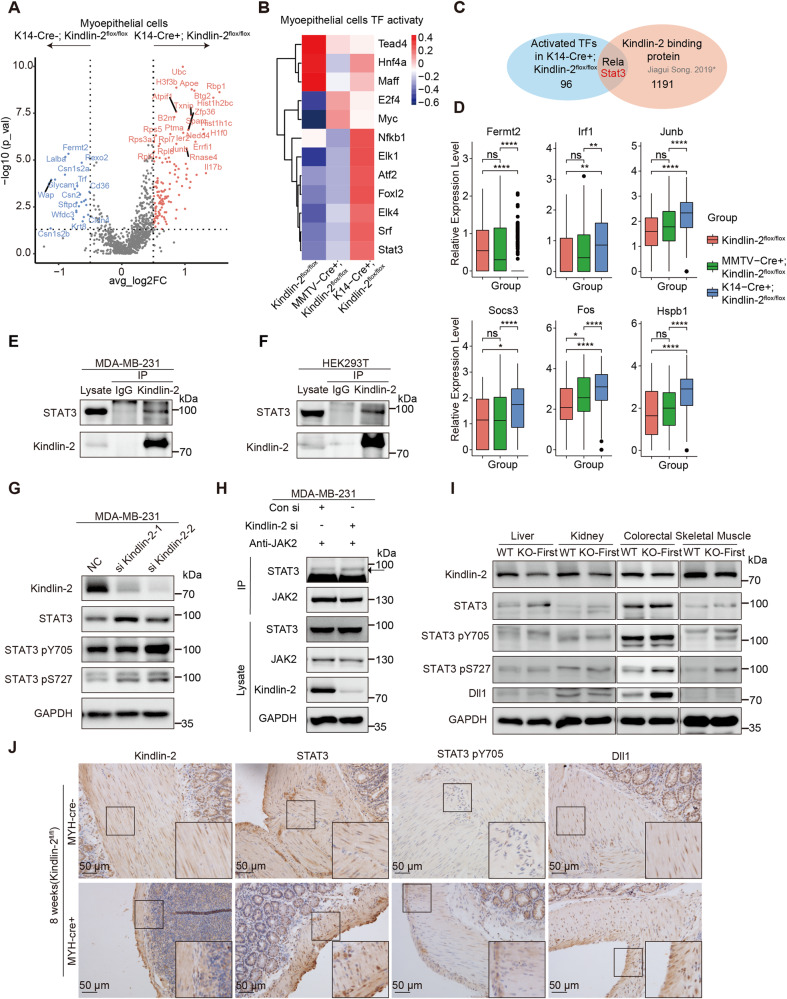
Kindlin-2 interacts with and inactivates STAT3 by competitively blocking JAK2/STAT3 interaction. **A** Volcano plot showing differential expression genes of myoepithelial cells between K14-Cre + ; Kindlin-2^flox/flox^ group and K14-Cre-; Kindlin-2^flox/flox^ group. Absolute (Log2(foldchange)) >0.5, *P* value < 0.05. **B** Heatmap showing relative transcription factor activity among three groups. **C** Venn Diagram showing the intersection of relative activated transcription factors in K14-Cre + ; Kindlin-2^flox/flox^ myoepithelial cell and Kindlin-2 binding protein. **D** Boxplot showing the mRNA expression levels of Stat3 canonical target genes and Kindlin-2 in myoepithelial cells from each group. Statistical testing was performed by unpaired wilcox-test. *P < 0.05, **P < 0.01, ***P < 0.001. **E** Co-immunoprecipitation assays were performed using lysates from MDA-MB-231 cells with control immunoglobulin G (IgG) or anti-Kindlin-2 antibody, followed by immunoblotting with an anti-STAT3 antibody. **F** Co-immunoprecipitation assays were performed using lysates from HEK293T cells with control immunoglobulin G (IgG) or anti-Kindlin-2 antibody, followed by immunoblotting with an anti-STAT3 antibody. **G** Western blot analysed of STAT3, STAT3 pY705, and STAT3 pS727 using specific antibodies through loss of function of Kindlin-2 in the cell lines: MDA-MB-231. GAPDH acted as an internal reference. **H** Control and Kindlin-2 siRNAs were transiently transfected into MDA-MB-231 cells separately. After 72 h, total protein extraction was prepared and a coIP assay was performed using anti-JAK2 antibody. Western blot was performed to detect STAT3 and JAK2 using specific antibodies. GAPDH acted as an internal reference. **I** Western blot showing the expression of Kindlin-2, STAT3, STAT3 pY705, STAT3 pS727, and Dll1 in liver, kidney, colorectal, and skeletal muscle in Kindlin-2 knockout-first (KO-First) and control mice (WT). **J** Immunohistochemical staining for Kindlin-2, STAT3, STAT3 pY705, and Dll1expression at the age of 8 weeks in Kindlin-2^fl/fl^; MYH-cre+ and Kindlin-2^fl/fl^; MYH-cre- control mice. Scale bar, 50 µm.

To identify the key molecular mechanisms underlying the function of Kindlin-2 in the mammary gland, we compared transformation of myoepithelial cells between the K14-Cre + ; Kindlin-2^flox/flox^ and Kindlin-2^flox/flox^ groups. We analyzed differentially expressed genes between these two groups (Fig. 5A) but did not directly identify factors explaining the regulatory process involving Kindlin-2 and Dll1. Kindlin-2 plays a role as an intracellular scaffold in multiple cellular processes [26, 27]; therefore, we examined whether Kindlin-2 could directly bind to and impact transcription factors to influence Dll1 expression. We used DoRothEA software to estimate transcription factor activities (Fig. 5B). In total, 96 transcription factors had activities that were higher in the myoepithelial cells of K14-Cre + ; Kindlin-2^flox/flox^ mice compared with MMTV-Cre + ; Kindlin-2^flox/flox^ and Kindlin-2^flox/flox^ mice [28]. Combining these findings with the results described above, two of those 96 transcription factors, namely Stat3 and Rela, may possess the ability to bind Kindlin-2 (Fig. 5C) [29].

Stat3 is one of a few reported transcription factors that directly regulate the expression of Dll1 [30]. Previous research indicated that Jak2–Stat3 activation was essential for Dll1 expression in neural precursor cells; in this process, Stat3 directly binds to the promoter of Dll1, enhancing its transcription. As the same FRMD domain is present in Kindlin-2 and the Jak family [31, 32], we speculated that Kindlin-2 might regulate the phosphorylation and activation of Stat3 by disrupting the interaction and catalytic reaction of Jak and Stat3. We checked the expression levels of Stat3 canonical target genes, such as *Irf1*, *Junb*, *Socs3*, *Fos*, and *Hspb1*, and found that they were significantly higher in myoepithelium of K14-Cre + ; Kindlin-2^flox/flox^ mammary gland, which is consisted with previous analysis of transcription factor activity (Fig. 5D). We confirmed that endogenous Kindlin-2 could interact with Stat3 in MDA-MB-231 and HEK293T cells (Fig. 5E, F). However, knockdown of Kindlin-2 promoted Stat3 phosphorylation at the Y705 and S727 sites, both of which are important for the activation of Stat3 in MDA-MB-231 cells (Fig. 5G), but not in HEK293T cells (Supplementary Fig. 7A, B). We assume that the regulatory interaction between Kindlin-2 and Stat3 may occur mainly in myoepithelial and basal cells, consistent with previous analysis that knockout of Kindlin-2 in MMTV-Cre + ; Kindlin-2^flox/flox^ mice did not interrupt Stat3 activation. Deletion of Kindlin-2 enhanced the amount of Stat3 co-immunoprecipitated with anti-JAK2 antibody in MDA-MB-231 cells (Fig. 5H).

To determine whether depletion of Kindlin-2 promotes Stat3 phosphorylation and expression of Dll1 through a common mechanism in smooth muscle tissue, we obtained liver, kidney, colorectal, and skeletal muscle tissue from Kindlin-2 knockout-first (KO-First), and WT mice. In the colorectal tissue, which contains abundant smooth muscle in the muscular layer, the levels of Dll1 expression and Stat3 phosphorylation were significantly elevated in Kindlin-2 knockout-first (KO-First) group (Fig. 5I). Furthermore, we detected Stat3 Y705 phosphorylation and Dll1 in Kindlin-2^fl/fl^; MYH-cre+ mice, whose Kindlin-2 was specifically knocked out in smooth muscle upon tamoxifen injection. Both Stat3 phosphorylation sites and Dll1 expression showed higher levels in colorectal tissue of Kindlin-2^fl/fl^; MYH-cre+ mice (Fig. 5J).

### Loss of Kindlin-2 activates Stat3 and upregulates Dll1 in the myoepithelium

**Fig. 6:**
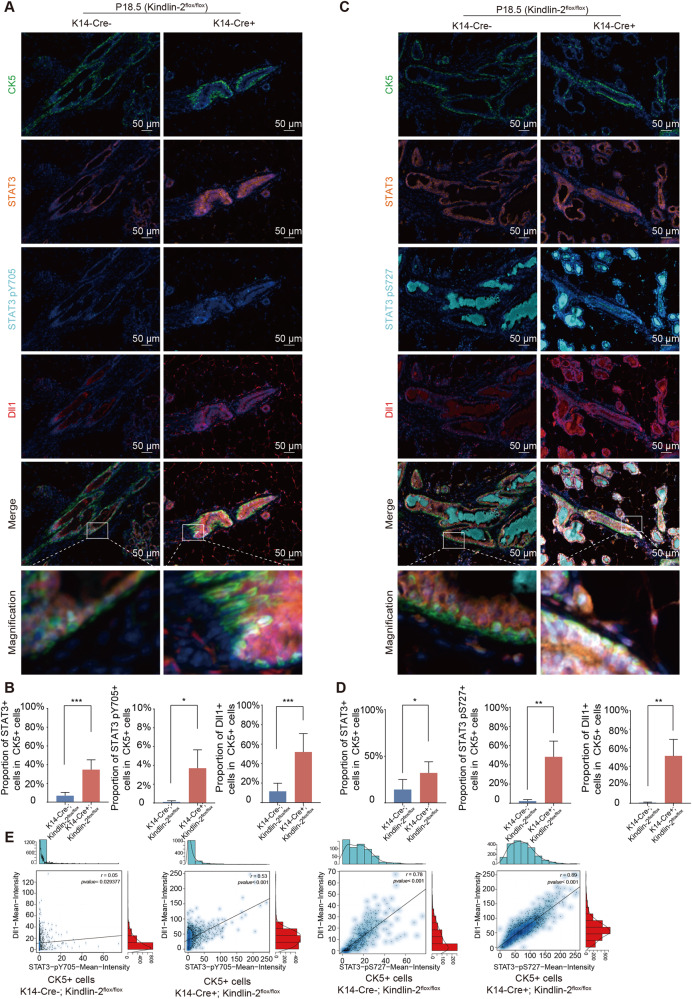
Loss of Kindlin-2 activates STAT3 and upregulates Dll1 in myoepithelium. **A** Multi-color staining showing CK5 (green), STAT3 (orange), STAT3 pY705 (cyan), Dll1 (red), and DAPI (blue) of P18.5 mammary gland in K14-Cre + ; Kindlin-2^flox/flox^ or K14-Cre-; Kindlin-2^flox/flox^ females. **B** Statistical analysis showing the proportion of STAT3+ cells in CK5+ cells, STAT3 pY705+ cells in CK5+ cells, and Dll1+ cells in CK5+ cells at P18.5 in K14-Cre + ; Kindlin-2^flox/flox^ group compared with K14-Cre-; Kindlin-2^flox/flox^ group (*n* = 5 fields of view at random per genotype). Statistical testing was performed by unpaired *t* test. Data are presented as mean values ± SD. *P < 0.05, **P < 0.01, ***P < 0.001. **C** Multi-color staining showing CK5 (green), STAT3 (orange), STAT3 pS727 (cyan), Dll1 (red), and DAPI (blue) of P18.5 mammary gland in K14-Cre + ; Kindlin-2^flox/flox^ or K14-Cre-; Kindlin-2^flox/flox^ females. **D** Statistical analysis showing the proportion of STAT3+ cells in CK5+ cells, STAT3 pS727+ cells in CK5+ cells, and Dll1+ cells in CK5+ cells at P18.5 in K14-Cre + ; Kindlin-2^flox/flox^ group compared with K14-Cre-; Kindlin-2^flox/flox^ group (*n* = 5 fields of view at random per genotype). Statistical testing was performed by unpaired *t* test. Data are presented as mean values ± SD. *P < 0.05, **P < 0.01. **E** Scatter plot showing the distribution and Pearson’s correlation of Dll1 and STAT3 pY705/pS727 fluorescence intensity in CK5+ cells from multiple fluorescence strain.

To confirm the regulatory interaction between Kindlin-2 and Stat3 in the mammary gland microenvironment in situ, we used multi-color immunofluorescence staining to qualify the phosphorylation levels of Stat3 in myoepithelial cells of the K14-Cre + ; Kindlin-2^flox/flox^ and Kindlin-2^flox/flox^ groups. When marked with CK5, myoepithelial cells exhibit a relatively high expression level of Stat3, with significantly elevated phosphorylation levels at the Y705 and S727 sites (Fig. 6A–D and Supplementary Fig. 5C, D). Interestingly, the phosphorylation levels of Y705 and S727 showed a consistent correlation with Dll1 expression, especially in myoepithelial cells from the K14-Cre + ; Kindlin-2^flox/flox^ group (Fig. 6E). These results demonstrate that loss of Kindlin-2 in myoepithelial cells induced the phosphorylation of Stat3, and the phosphorylated Stat3 functioned as an activated transcription factor to induce the expression of Dll1 and thus regulate Notch pathway activation in luminal cells in situ.

From these results, we determined that Kindlin-2 is an essential regulator in myoepithelial cells that controls the maturation of luminal cells through the Jak2–Stat3–Dll1–Notch1 axis.

### Tangeretin treatment improves the lactation function of K14-Cre + ; Kindlin-2^flox/flox^ female mice

**Fig. 7:**
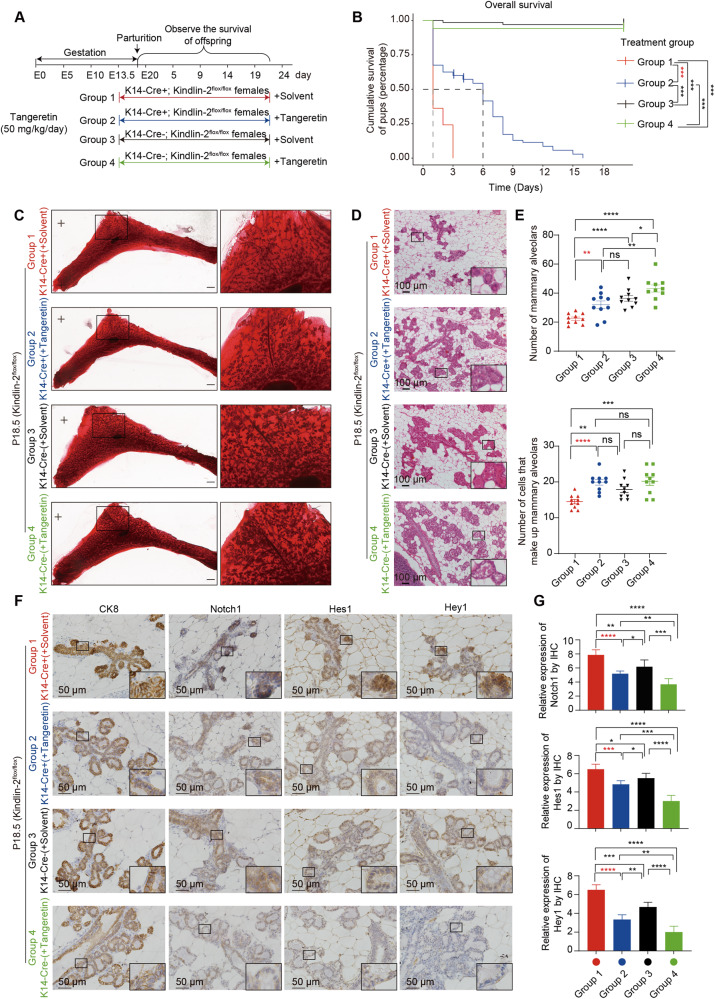
Inhibitor of Notch signaling pathway tangeretin treatment can improve the lactation function of K14Cre + ; Kindlin-2^flox/flox^ female mice. **A** Therapeutic schedule of tangeretin treatment on K14-Cre + ; Kindlin-2^flox/flox^ and K14-Cre-; Kindlin-2^flox/flox^ female mice. Eighteen K14-Cre + ; Kindlin-2^flox/flox^ female mice weighing 20–25 g were divided into 2 groups at random. The mice caged mating with K14-Cre-; Kindlin-2^flox/flox^ male mice is recorded as E0. Group 1: nine K14-Cre + ; Kindlin-2^flox/flox^ female mice were treated with solvent 100 µl from E13.5 and equivalent solvent were used every day. Group 2: nine K14-Cre + ; Kindlin-2^flox/flox^ female mice were treated with tangeretin 50 mg/kg from E13.5 and equivalent drugs were used every day. Group 3: nine K14-Cre-; Kindlin-2^flox/flox^ female mice were treated with solvent 100 µl from E13.5 and equivalent solvent were used every day. Group 4: nine K14-Cre-; Kindlin-2^flox/flox^ female mice were treated with tangeretin 50 mg/kg from E13.5 and equivalent drugs were used every day. The offsprings of these mice was observed and the survival status was recorded. **B** K-M plot analyses were performed to uncover the difference of survival rate of these offsprings between Tangeretin treatment groups and control groups (Group 1: *n* = 36, Group 2: *n* = 40, Group 3: *n* = 30, Group 4: *n* = 34). Statistical testing was performed by Log-Rank test. *P < 0.05, **P *<* 0.01, ***P *<* 0.001. **C** Whole-mounted staining of P18.5 mammary gland in K14-Cre + ; Kindlin-2^flox/flox^ treatment groups or K14-Cre-; Kindlin-2^flox/flox^ treatment groups. **D** HE sections staining of P18.5 mammary gland in K14-Cre + ; Kindlin-2^flox/flox^ treatment groups or K14-Cre-; Kindlin-2^flox/flox^ treatment groups. Scale bars, 100 µm. **E** Statistical analysis showing the number of mammary alveolars and cells that make up mammary alveolars in K14-Cre + ; Kindlin-2^flox/flox^ treatment groups compared with K14-Cre-; Kindlin-2^flox/flox^ treatment groups (*n* = 10 fields of view at random per treatment group). Statistical testing was performed by unpaired *t* test. Data are presented as mean values ± SEM. *P < 0.05, **P < 0.01, ***P < 0.001, ****P < 0.0001. **F** Immunohistochemical staining for CK8, Notch1, Hes1 and Hey1 expression of P18.5 mammary gland in four groups. Scale bar, 50 µm. **G** Quantification for the Notch1 levels in luminal cells (CK8 positive), the target protein of Notch signaling pathway Hes1 and Hey1 levels in the ducts of four groups (*n* = 6). Statistical testing was performed by unpaired *t* test. Data are presented as mean values ± SD. *P < 0.05, **P < 0.01, ***P < 0.001, ****P < 0.0001.

Absence of Kindlin-2 in the myoepithelium activates Stat3 and sharply increases communication along the Notch pathway between myoepithelial cells and luminal progenitors. Compared with the extensive distribution of phosphorylated Stat3, the relatively precise expression of Notch1 in luminal and alveolar progenitor cells provides a better potential drug target. Herein, we assumed that targeting Notch activation with an effective Notch1 inhibitor, tangeretin [33, 34], would rescue the dysfunction and empower differentiation of the naïve luminal epithelium. We divided the pregnant female mice of Kindlin-2^flox/flox^ and K14-Cre + ; Kindlin-2^flox/flox^ into four groups: (1) K14-Cre + ; Kindlin-2^flox/flox^ pregnant mice treated with solvent beginning at E13.5; (2) K14-Cre + ; Kindlin-2^flox/flox^ pregnant mice treated with tangeretin at 50 mg/kg/day beginning at E13.5; (3) K14-Cre-; Kindlin-2^flox/flox^ pregnant mice treated with solvent beginning at E13.5; and (4) K14-Cre-; Kindlin-2^flox/flox^ pregnant mice treated with tangeretin at 50 mg/kg/day beginning at E13.5 (Fig. 7A). Consistent with our hypothesis, treatment with tangeretin partially restored the nursing ability of K14-Cre + ; Kindlin-2^flox/flox^ dams (Fig. 7B). The histology of the mammary gland from those four groups showed that alveologenesis was partly rescued with tangeretin treatment in the K14-Cre + ; Kindlin-2^flox/flox^ group (Fig. 7C–E). To verify that tangeretin inhibits luminal epithelium Notch pathway, we detected the expression levels of Notch1, Hes1, and Hey1 in four group (Fig. 7F–G). The Immunohistochemical staining showed that Notch pathway was significantly inhibited in the K14-Cre + ; Kindlin-2^flox/flox^ group upon treatment with tangeretin.

These results suggested that tangeretin treatment blocked abnormal activation of the Notch pathway in luminal cells induced by loss of Kindlin-2 in myoepithelial cells, supporting luminal epithelium function in lactation.

## Discussion

**Fig. 8:**
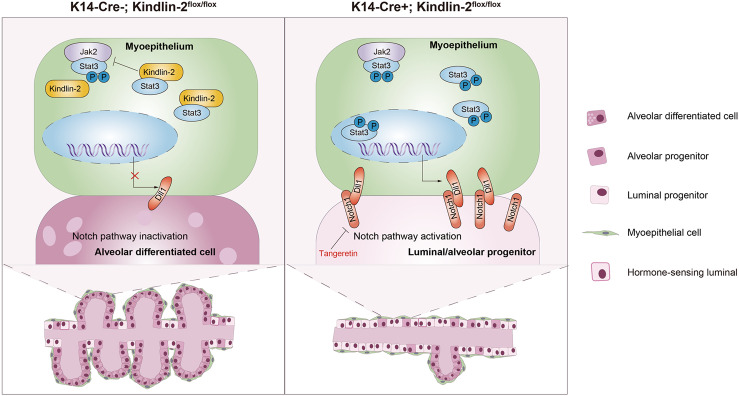
A working model. Depletion of Kindlin-2 in myoepithelium activates the Jak2–Stat3–Dll1 axis, which activates Notch signaling in luminal progenitors and inhibits theirs differentiation and maturation during gestation.

Researchers have made great efforts to reconstruct the trajectory of mammary gland development and its disequilibrium associated with cancer [35–38]. Most research has focused on the physiological or pathological state of the luminal epithelium, which is considered the most important factor affecting mammary gland function. With deepening research into the mammary gland, more attention is being paid to its microenvironment, including cross-talk among immune cells, stromal cells, epithelial cells, and the extracellular matrix, rather than focusing solely on luminal epithelial cells, by combining a fairly comprehensive model with high-resolution methods [39]. In this study, we demonstrate that Kindlin-2 is a critical regulator located in the myoepithelium that prevents the phosphorylation of Stat3 and expression of Dll1 to preserve the order of luminal progenitor maturation during gestation (Fig. 8A). Our findings also indicate that the regulatory axis of Kindlin-2–Stat3–Dll1 is common to smooth muscle-enriched tissues. Through targeting Notch1 with tangeretin, we partially reversed the retardation of mammary luminal cells caused by the loss of Kindlin-2 in myoepithelial cells.

Myoepithelium is a specialized muscle-like differentiated epithelium [40, 41]. Many difficulties arise in investigations of the cross-talk between the myoepithelium and luminal epithelium. One such difficulty is that highly terminal differentiated myoepithelial cells are present in low proportions and have no stable cell lines. Most research has been performed with genetically modified mouse models to capture in vivo mechanisms [42, 43]. However, many myoepithelium-specific mechanisms can only be revealed at single-cell resolution due to the distinct histological pattern of the myoepithelium, a layer of cells that is very thin and clings to luminal epithelial cells. Therefore, previous research using bulk sequencing or total lysis might have been contaminated with other cells, concealing key mechanisms. Using single-cell RNA-seq, we successfully determined the mechanism through which loss of Kindlin-2 induces overactivation of Stat3, a transcription factor that is reported to regulate Dll1 expression, and demonstrated that Kindlin-2 could impact the activation of Stat3 through binding with Jak2 and Stat3 in the basal-like cell line MDA-MB-231. We also used multiple-color staining to demonstrate the impact of Stat3 and Dll1 after Kindlin-2 depletion in myoepithelial cells in situ and confirmed this mechanism in smooth muscle tissues of Kindlin-2 knockout mice. The important role of Kindlin-2 in Jak2–Stat3 signaling is specific to smooth muscle and muscle-like cell lineages and is not present in the luminal cells, epithelial cells (HEK293T), or other tissues with a low abundance of smooth muscle.

Numerous questions remain to be resolved, such as how the dynamic regulation of both Notch pathway ligands and receptors occurs throughout the development and pregnancy cycle, as signaling activation does not affect only the recipient cells. Deficiency of Dll1 in basal cells leads to a more complete block of development of mammary ducts, and the expression of Dll1 in mammary gland stem cells (MaSCs) can activate the Notch pathway in macrophages, which promote the stemness of MaSCs through Wnt signaling [44]. However, our data did not show this type of regulation between myoepithelial cells and macrophages, which might be due to the specific focal periods of this study. Aside from Dll1, the Notch ligand Jag1 in basal cells can also receive Robo1 and β-catenin signaling regulation, mediating luminal progenitor maturation through the Notch pathway [45]. Our findings suggest that at 14.5 days of pregnancy, the luminal epithelium produces alveolar progenitors with Notch1 expression, and thus we applied tangeretin in dams since the later trimester of pregnancy.

During mammary gland development and maturation, the main process known to regulate mammary duct development and alveologenesis is the variation of several hormones, including estrogen, progestin, and prolactin [46]. Previous research reported that a high level of estrogen could protect Dll1 from the ubiquitin proteasomal degradation pathway in breast cancer [47]. The expression levels of receptors and ligands of the Notch signaling pathway changed dynamically and were consistent with the levels of progesterone and estradiol in the mouse oviduct during the estrous cycle [48]. Another study reported that estrogen could improve the proliferation and differentiation of bone marrow stem cells through the Notch signaling pathway [49]. Whether a similar regulatory network exists between hormones and the Notch pathway during mammary gland development and the detailed regulation mechanisms of Notch signaling ligands and receptors during the hormonal cycle remain to be resolved.

Activation of Stat3 in the luminal epithelium is associated with cell death and modulates the inflammatory microenvironment during involution [50]. As a common inflammatory disease during lactation, mastitis reduces milk supply. Previous research noted that the activation Il6–Jak2–Stat3 axis is associated with plasma cell mastitis [51]. Our study further elucidates the function of Stat3 in Notch pathway regulation. Whether the activation of Stat3 in myoepithelium, triggered by Il6 and interferon, in mothers with mastitis, is the etiology of lactation failure via cross-cell signal transduction requires further investigation.

Based on the efficacy of tangeretin treatment in our research, we successfully constructed a mouse model of lactation disorder driven by abnormal activation of the Notch signaling pathway, which can be broadly applied in further molecular mechanism and drug discovery research. The application of tangeretin might benefit mothers who are unable to feed their infants due to abnormality of the Notch pathway and Jak2–Stat3 signaling.

Fully demonstrating the regulation of intercellular signaling and intracellular transduction in mammary gland development and cancer is a worthwhile topic for future research. Further investigations are required to determine whether the regulatory interactions between Kindlin-2 and the Jak2–Stat3–Dll1 axis are common across cell lineages with high levels of Kindlin-2, such as myoepithelial cells, basal cells, and fibroblast cells. Whether the complex regulatory processes among Kindlin-2, the Jak–Stat signaling pathway (related to inflammation), and the Notch signaling pathway (associated with cell stemness and fate differentiation) are involved in breast-related diseases such as mastitis and breast cancer is another important topic of further investigation.

## Methods

### Mice and genotyping

All animal experiments were approved by the Peking University Animal Care and Use Committee. We bought Kindlin-2 knockout-first mice (ID: 38007, C57BL/6 genetic backgroud) from International Knockout Mouse Consortium (IKMC). Our lab developed the Kindlin-2 floxed mice. LoxP sites flanked the fifth and sixth exons of *FERMT2*. We bought Kindlin-2^LSL/LSL^ transgenic mice from Shanghai Model Organisms Center, Inc. MMTV-Cre+ transgenic mice were bought from Nanjing Biomedical Research Institute of Nanjing University. K14-Cre+ mice were provided by Zhengquan Yu lab in China Agricultural University. Kindlin-2 floxed (Kindlin-2^flox/flox^) mice were crossed with K14-Cre+ and MMTV-Cre+ mice to generate Kindlin-2 heterozygous mice (K14-Cre + ; Kindlin-2^flox/wt^ and MMTV-Cre + ; Kindlin-2^flox/wt^), which were further backcrossed with Kindlin-2^flox/flox^ mice to generate mammary myoepithelial-specific and luminal epithelium-specific Kindlin-2 knockout mice. Kindlin-2 overexpressed (Kindlin-2^LSL/LSL^) mice were crossed with K14-Cre+ to generate Kindlin-2 heterozygous mice (K14-Cre + ; Kindlin-2^LSL/wt^), which were further backcrossed with Kindlin-2^LSL/LSL^ mice to generate mammary myoepithelial-specific Kindlin-2 knock-in mice. Kindlin-2^fl/fl^; MYH-cre+ mice were described previously [4]. Genotyping was determined via PCR using primers: Kindlin-2 knockout-first: P1 5ʹ- TACAGGTGGCTGACAAGATCC -3ʹ, P2 5ʹ- GTGAGGCTCACCTTTCAGAGG -3ʹ, P3 5ʹ- CAACGGGTTCTTCTGTTAGTCC -3ʹ; Kindlin-2: forward 5ʹ- TACAGGTGGCTGACAAGATCC -3ʹ, reverse 5ʹ- GTGAGGCTCACCTTTCAGAGG -3ʹ; Cre: forward 5ʹ- ATTTGCCTGCATTACCGGTCG -3ʹ, reverse 5ʹ- CAGCATTGCTGTCACTTGGTC -3ʹ; Kindlin-2^LSL/LSL^: P1 5ʹ- TCAGATTCTTTTATAGGG-GACACA -3ʹ, P2 5ʹ- TAAAGGCCACTCAATGCTCACTAA -3ʹ, P3 5ʹ- GGTGTTGTCGGGG-AAATCATCGTC -3ʹ, P4 5ʹ- AGGAGCCTGCCAAGTAAC -3ʹ; MYH-cre: SMWT1 5ʹ- TGACCCCATCTCTTCACTCC -3ʹ, SMWT2 5ʹ- AACTCCACGACCACCTCATC -3ʹ, phCREAS1 5ʹ- AGTCCCTCACATCCTCAGGTT -3ʹ.

### Dosing experiment

We divided the pregnant female mice from K14-Cre-; Kindlin-2^flox/flox^ and K14-Cre + ; Kindlin-2^flox/flox^ into 4 groups: (1) K14-Cre + ; Kindlin-2^flox/flox^ pregnant mice were treated with solvent by oral gavage from E13.5 and equivalent solvent were used every day. (2) K14-Cre + ; Kindlin-2^flox/flox^ pregnant mice were treated with Tangeretin (Bi De Yi Yao, BD33755), a Notch1 inhibitor, 50 mg/kg/day by oral gavage from E13.5 and equivalent drugs were used every day. (3) K14-Cre-; Kindlin-2^flox/flox^ pregnant mice were treated with solvent by oral gavage from E13.5 and equivalent solvent were used every day. (4) K14-Cre-; Kindlin-2^flox/flox^ pregnant mice were treated with Tangeretin 50 mg/kg/day by oral gavage from E13.5 and equivalent drugs were used every day.

### Cell culture

Human breast cancer (MDA-MB-231) and human embryonic kidney (HEK293T) cell lines were cultured in DMEM medium containing 10% FBS (Invitrogen, Carlsbad, CA, USA), supplemented with 100 units/ml penicillin and 100 mg/ml streptomycin. These cell lines were purchased from ATCC. All cells were cultured at 37 °C in 5% CO_2_. According to the manufacturer’s instructions (Invitrogen), 50%–70% of confluent cells were transfected with indicated siRNA using Lipofectamine RNAiMAX for transient transfection. Specific siRNA targeting human Kindlin-2 (Kindlin-2 siRNA) was designed by Guangzhou Rui Bo (China). Kindlin-2si_001: AAGCUGGUGGAGAAACUCG; Kindlin-2si_002: CAGCGAGAAUCUUGGAGGC.

### Western blot analysis

Cell lysates or tissues were treated using RIPA lysis buffer supplemented with 1× inhibitor cocktail (Roche) and 1× Roche PhosSTOP phosphatase inhibitor cocktail (Roche). Aliquots of 30 μg protein were separated by SDS-PAGE and transferred to the polyvinylidene fluoride (PVDF) membrane. Membranes were blocked in 5% non-fat powdered milk at room temperature for 1 h and incubated with specific antibodies overnight at 4 °C. Antibodies used for western blot analysis in this study were: rabbit anti-Kindlin-2 (Sigma, K3269, 1:1000 dilution); mouse anti-GAPDH (Zhong Shan Jin Qiao, TA-08, 1:1000 dilution); mouse anti-tubulin (Sigma, clone B-5-1-2, 1:1000 dilution); rabbit anti-DDDDK tag (Abcam, ab205606, 1:1000 dilution); rabbit anti-STAT3 (Abcam, ab68153, 1:1000 dilution); rabbit anti-pSTAT3-S727 (Abcam, ab32143, 1:1000 dilution); rabbit anti-pSTAT3-Y705 (Abcam, ab76315, 1:1000 dilution); rabbit anti-Dll1 (Abcam, ab10554, 1:1000 dilution); rabbit anti-JAK2 (Abcam, ab108596, 1:1000 dilution). After washing three times in TBST buffer, the membranes were incubated with appropriate HRP-conjugated secondary antibodies at room temperature for 1 h. The Super Signal Chemiluminescence kit (Thermo Fisher Scientific) was used to detect immobilized antibodies.

### Immunoprecipitation

Immunoprecipitation methods were described previously [52]. MDA-MB-231 and HEK293T cell lysates were treated using RIPA lysis buffer supplemented with 1× inhibitor cocktail (Roche) and 1× Roche PhosSTOP phosphatase inhibitor cocktail (Roche). After centrifugation at 12,000 rpm for 15 min at 4 °C, cell debris was removed. Precleared lysates were incubated with indicated antibodies and normal IgG (as controls) overnight at 4 °C. 40 μl of protein A or protein G agarose (Santa Cruz Biotechnology, Inc.) were incubated with Lysates with rotation at 4 °C for 6 h. The complex was washed with RIPA buffer five times, resuspended with 60 μl 2× loading buffer, and cooked at 100 °C for 8 min, which was detected by western blot analysis.

### Histology and immunohistochemistry

Mouse mammary glands were fixed with 4% buffered formalin (Fisher), embedded in paraffin, and sectioned at 5 μm before staining. The prepared sections were stained with immunohistochemistry to detect different proteins or with H&E to examine the structure. Briefly, sections were deparaffinized with xylene and rehydrated with ethanol. 3% hydrogen peroxide was used to block the sections for 15 min at room temperature. After washing in phosphate buffer saline (PBS, pH 7.6) three times, antigen-retrieval was performed by heating slides in 0.01 M citrate buffer (pH 6.0) for 20 min in a 100 °C water bath. When slides cooled at room temperature, using 10% normal goat serum blocked the non-specific binding of antibodies for 20 min. The sections were probed with anti-Kindlin-2(Sigma, K3269, 1:100 dilution), anti-Dll1 (Abcam, ab10554, 1:200 dilution), anti-Hes1 (Abcam, Ab71559, 1:200 dilution), anti-Notch1 (EPITOMICS, Cat#1935-1, 1:200 dilution), anti-STAT3 (Abcam, ab68153, 1:200 dilution); rabbit anti-pSTAT3-Y705 (Abcam, ab76315, 1:100 dilution), anti-CK5 (Abcam, ab52635, 1:200 dilution), or anti-CK8 (Abcam, ab53280, 1:400 dilution) overnight. After washing in PBS three times, the sections were incubated with biotinylated IgG (Zhongshanjinqiao, PV6001) for 60 min at room temperature. After washing in PBS three times, Diaminobenzidine (DAB) was used as a substrate, and hematoxylin was used as a counterstain. A BX51 microscope (Olympus) was used to examine and photograph for immunostainings.

### Whole-mounting of mammary glands

Mammary glands were fixed in Carnoy’s solution containing 10% glacial acetic acid, 30% chloroform, and 60% ethanol for at least 24 h at room temperature. The sections were rehydrated with ethanol and then stained with carmine solution (HARVEYBIO, C21940-1G) overnight at room temperature.

### Quantitative analysis of tissue section image

Performed multi-color staining according to the kit instructions (TissueGnostics, TGFP550). Using StrataQuest v7.0.158 software (TissueGnostics, Vienna, Austria) performed Immunofluorescence image analysis. Total cells were identified based on the DAPI staining.

### Statistical analysis

Using GraphPad Prism 9.0 software performed statistical analysis. All the results are expressed as the mean values ± SEM or mean values ± SD. Using unpaired one- or two-tailed Student’s *t* tests performed quantitative PCR analysis. Statistical significance was defined as *p* < 0.05. All data statistics were tested for homogeneity of variance, and then we chose whether to do a nonparametric test or a parametric test.

### Tissue dissociation and preparation

The fresh mammary gland tissues were washed with Hanks Balanced Salt Solution (HBSS) for 3 times and minced into 1–2 mm pieces. Then the tissue pieces were digested with 2 ml GEXSCOPE® Tissue Dissociation Solution (Singleron) at 37 °C for 15 min in 15 ml centrifuge tube with sustained agitation. After digestion, using 40-micron sterile strainers to filter the samples and centrifuging the samples at 1000 rpm for 5 min. Then the supernatant was discarded, and the sediment was resuspended in 1 ml PBS (HyClone). To remove the red blood cells, 2 mL GEXSCOPE® red blood cell lysis buffer (Singleron) was added at 25 °C for 10 min. The solution was then centrifuged at 500 × *g* for 5 min and suspended in PBS. Cells were incubated with 7-AAD (1:1000, Invitrogen) on ice for 30 min, and sorted by flow cytometry to filter dead cells.

### Single cell RNA sequencing

Single-cell suspensions with 1 × 10^5^ cells/mL in concentration in PBS (HyClone) were prepared. Single-cell suspensions were then loaded onto microfluidic devices and scRNA-seq libraries were constructed according to Singleron CLindex® protocol by GEXSCOPE® Single-Cell RNA Library Kit (Singleron Biotechnologies). Mixture libraries were diluted to 4 nM and pooled for sequencing. Pools were sequenced on Illumina HiSeq X with 150 bp paired end reads.

### scRNA-seq quantifications and statistical analysis

Raw reads were processed and aligned to the genome mm10, to generate gene expression profiles using an internal pipeline. The Seurat program (http://satijalab.org/seurat/, R package, v.4.1.0) was applied for analysis of RNA-Sequencing data.

A total of 16,702 cells were sequenced (5230 from Kindlin-2^flox/flox^, 6,052 from MMTV-Cre + ; Kindlin-2^flox/flox^ and 5,420 from K14-Cre + ; Kindlin-2^flox/flox^). First, we removed low-quality cells by modeling mitochondrial to nuclear gene content to <10% and gene numbers higher than 1000 and lower than 4000, as a result 16,261 high quality cells (5,155 from Kindlin-2^flox/flox^, 5876 from MMTV-Cre + ; Kindlin-2^flox/flox^ and 5,230 from K14-Cre + ; Kindlin-2^flox/flox^) for downstream analysis. After normalization and scale the expression matrix, we selected 2000 high variable genes for PCA analysis. Cells were separated into 24 clusters, using the top 30 principal components and resolution parameter at 0.8. Further, we integrated our dataset with GSE106273 using Fast integration using reciprocal PCA function in Seurat.

### Differentially expressed genes (DEGs) analysis

Genes expressed in more than 50% of the cells in a cluster and with average log (Fold Change) of greater than 1 were selected as DEGs based on Wilcox likelihood-ratio test with default parameters.

### Cell type annotation

The main lineage identification of each cluster was determined with the expression of canonical markers of Epithelial cells (Epcam), immune cells (Ptprc) and stroma cells (Col1a1). The cell lineages were determined by SingleR (https://github.com/LTLA/SingleR. v.1.8.1) with MouseRNAseq Data as reference cell type from celldex (http://github.com/LTLA/celldex. v.1.4.0) and detailed cell markers from CellMarker (https://bio-bigdata.hrbmu.edu.cn/CellMarker/).

### Trajectory analysis

To map differentiation of cell subtypes during lactation, pseudotime trajectory analysis was performed with Monocle2. For constructing the trajectory, top 400 highly variable genes among 4 types of luminal epithelial cells, and dimension-reduction was performed by DDRTree.

### Cell-cell interaction

CellChat was used to perform cell-cell communication calculation and analysis for the mammary epithelial clusters. The CellChat results were used to reveal both incoming communication patterns of target cells and outgoing communication patterns of the secreting cells. To reveal the strength of specific pathways among mammary epithelial cells clusters, we selected the most relevant Notch signaling pathway for further visualization.

### Transcription factor regulatory analysis

Activity of the most variable transcription factors in the myoepithelial was determined using the DoRothEA (v1.8.0) algorithm for single-cell RNA-sequencing. Mouse regulons (grades A–C) were obtained from the DoRothEA R package. We extracted the 12 most variable transcription factors over groups. The predicted transcription factors were compared to their corresponding gene expression, and those with low real expression (normalized expression < 0.5) were removed from analysis.

### Supplementary information

Supplementary files

Original western blots

Reproducibility checklist

## Data Availability

The raw RNA-seq data used in this study are available in the Sequence Read Archive. The raw data and processed data of single-cell transcriptomic data generated in this study have been deposited in the Gene Expression Omnibus (GEO) database under accession code GSE222028. The publicly available single cell dataset used in this study are available from the Gene Expression Omnibus (accession numbers GSE106273). All the other data supporting the findings of this study are available within the article and its Supplementary Information files.

## Code availability

The code applied in this study is available upon request.
