# Supplementary material for: Kindlin-2 in myoepithelium controls luminal progenitor commitment to alveoli in mouse mammary gland
Source: Cell Death Dis. 2023 Oct 13;14(10):675. doi: 10.1038/s41419-023-06184-2 (PMC10576046; doi:10.1038/s41419-023-06184-2)
Supplement: Supplementary file 1 — Supplementary files [file 41419_2023_6184_MOESM1_ESM.docx]

Supplementary materials for

Kindlin-2 in myoepithelium controls luminal progenitor commitment to alveoli in mouse mammary gland

Zhenbin Wang^1#^, Lei Zhang^1#^, Bing Li^1, 2#^, Jiagui Song^1#^, Miao Yu^1^, Jing Zhang^1^, Ceshi Chen^3, 4^*, Jun Zhan^1^*, Hongquan Zhang^1^*


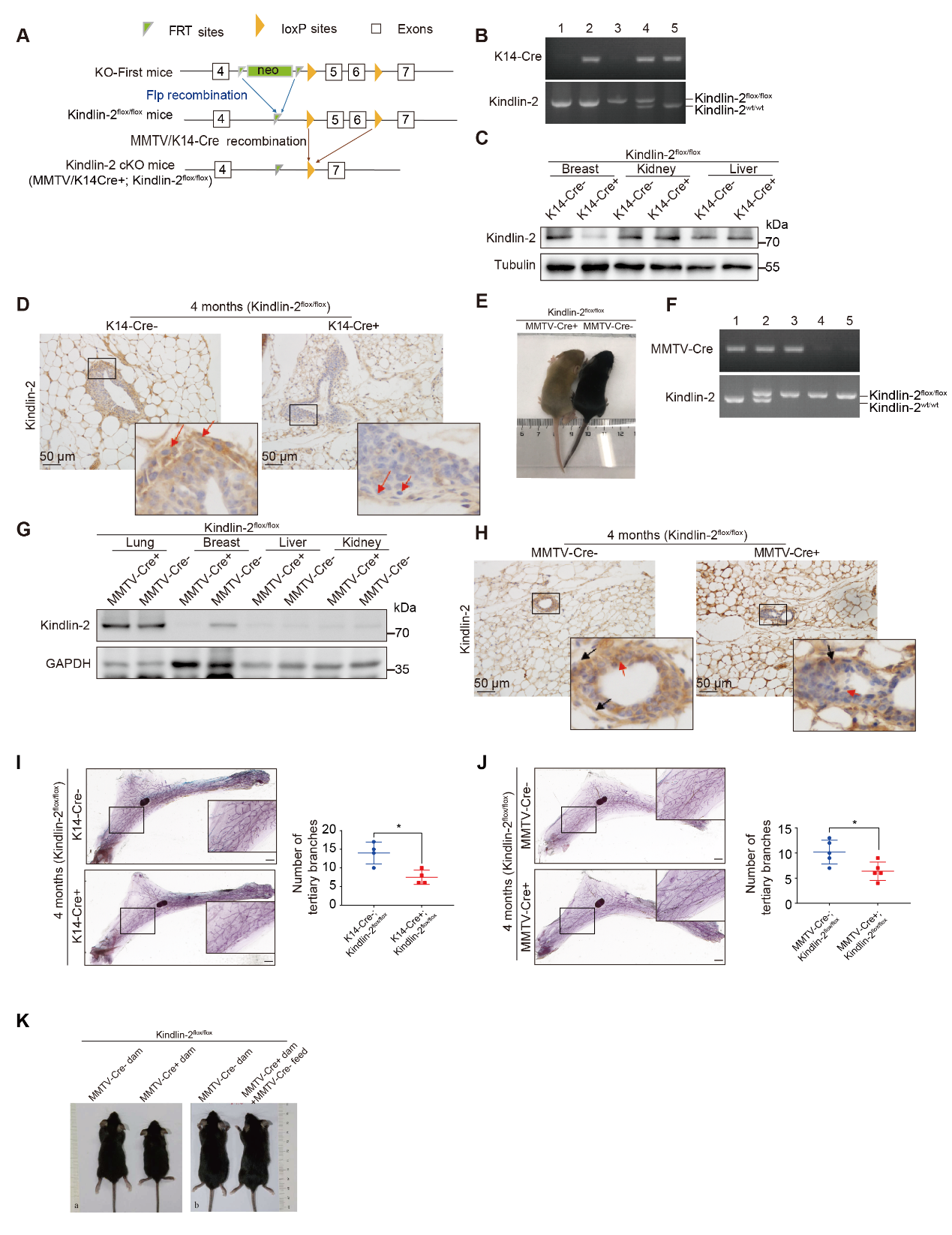
Fig S1. Generation of mammary luminal and myoepithelial cells specific knockout of Kindlin-2 mouse.

A) Schematic diagram of the construction of the mouse models.

B) Characterizing the genotype by polymerase chain reaction in K14-Cre+; Kindlin-2^flox/flox^ or K14-Cre-; Kindlin-2^flox/flox^ littermate control mice.

C) Western blot showing the expression of Kindlin-2 in liver, breast and kidney in K14-Cre+; Kindlin-2^flox/flox^ or K14-Cre-; Kindlin-2^flox/flox^ littermate control mice.

D) Immunohistochemical staining for Kindlin-2 expression in 4-month-old mammary gland of K14-Cre+; Kindlin-2^flox/flox^ and K14-Cre-; Kindlin-2^flox/flox^ females. Scale bar, 50 µm.

E) Distinguishing the genotype in MMTV-Cre+; Kindlin-2^flox/flox^ or MMTV-Cre-; Kindlin-2^flox/flox^ littermate control mice by mice coat color. Yellow represents MMTV-Cre+; Kindlin-2^flox/flox^ genotype and black represents MMTV-Cre-; Kindlin-2^flox/flox^ genotype.

F) Characterizing the genotype by polymerase chain reaction in MMTV-Cre+; Kindlin-2^flox/flox^ or MMTV-Cre-; Kindlin-2^flox/flox^ littermate control mice.

G) Western blot showing the expression of Kindlin-2 in lung, breast, liver and kidney in MMTV-Cre+; Kindlin-2^flox/flox^ or MMTV-Cre-; Kindlin-2^flox/flox^ littermate control mice.

H) Immunohistochemical staining for Kindlin-2 expression in 4-month-old mammary gland of MMTV-Cre+; Kindlin-2^flox/flox^ and MMTV-Cre-; Kindlin-2^flox/flox^ females. Scale bar, 50 µm.

I) Left: whole-mounted staining in 4-month-old mammary gland of K14-Cre+; Kindlin-2^flox/flox^ or K14-Cre-; Kindlin-2^flox/flox^ littermate control mice. Right: Statistical analysis showing the number of tertiary branches in K14-Cre+; Kindlin-2^flox/flox^ group compared with K14-Cre-; Kindlin-2^flox/flox^ group (*n*=4 fields of view at random per genotype). Statistical testing was performed by unpaired *t*-test. Data are presented as mean values+/− SD. **P* < 0.05, ***P* < 0.01, ****P* < 0.001.

J) Left: whole-mounted staining in 4-month-old mammary gland of MMTV-Cre+; Kindlin-2^flox/flox^ or MMTV-Cre-; Kindlin-2^flox/flox^ littermate control mice. Right: Statistical analysis showing the number of tertiary branches in MMTV-Cre+; Kindlin-2^flox/flox^ group compared with MMTV-Cre-; Kindlin-2^flox/flox^ group (*n*=5 fields of view at random per genotype). Statistical testing was performed by unpaired *t*-test. Data are presented as mean values+/− SD. **P* < 0.05, ***P* < 0.01, ****P* < 0.001.

K) Foster nursing experiment of MMTV-Cre-; Kindlin-2^flox/flox^ group feeding MMTV-Cre+; Kindlin-2^flox/flox^ group. (a) Mice in MMTV-Cre+; Kindlin-2^flox/flox^ group were lactated for two weeks by females in MMTV-Cre-; Kindlin-2^flox/flox^ group; (b) After weaning and feeding for 8 weeks, the body size difference of pups born in MMTV-Cre+; Kindlin-2^flox/flox^ group and MMTV-Cre-; Kindlin-2^flox/flox^ group.


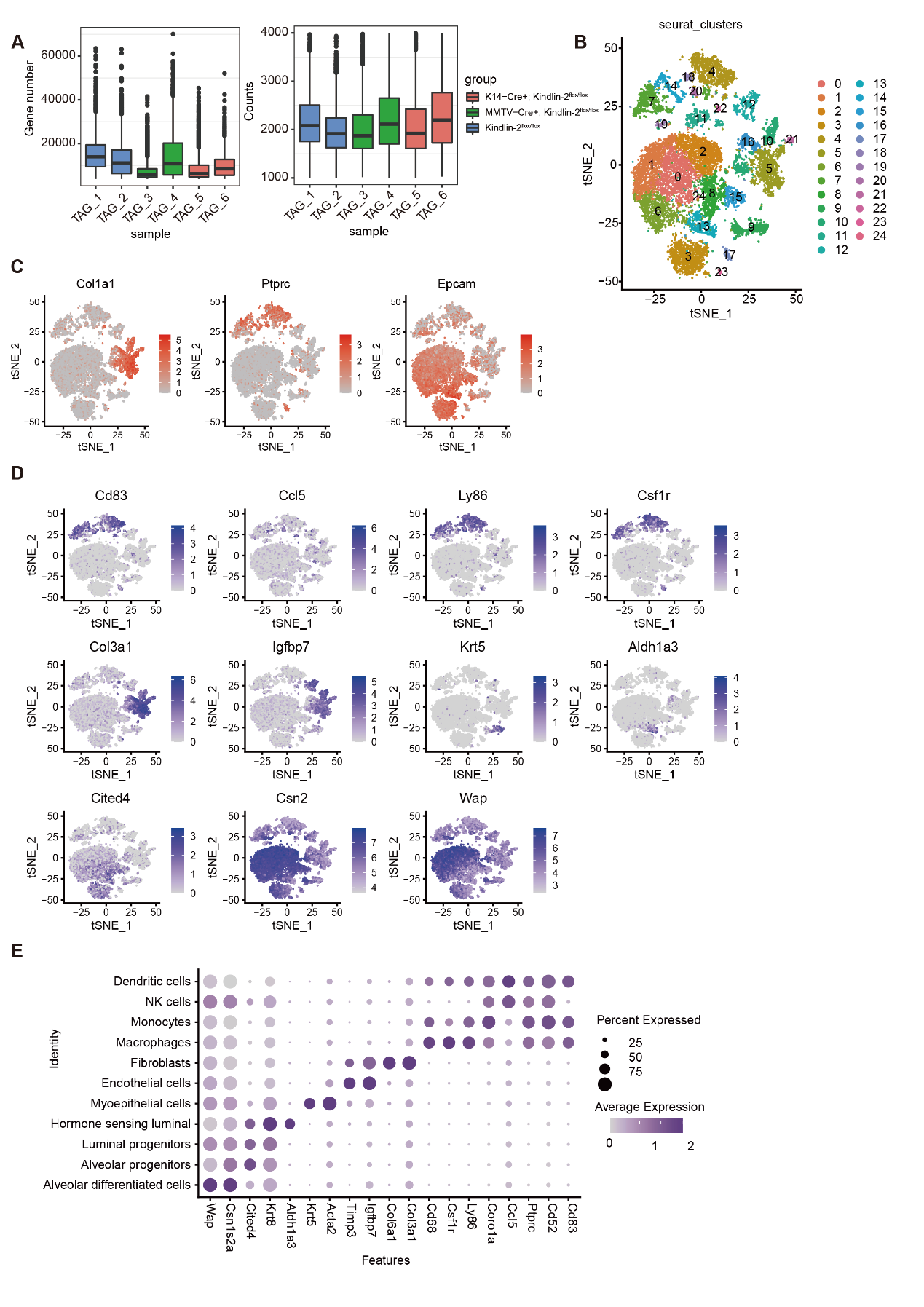


Fig S2. Distinct cell lineages determined by single-cell RNA-Seq analysis of Kindlin-2^flox/flox^, K14-Cre+; Kindlin-2^flox/flox^ and MMTV-Cre+; Kindlin-2^flox/flox^ pregnant mouse mammary glands.

1. Boxplot showing gene number and count distribution of 6 mammary glands sample from 3 genotypes mouse.
2. T-SNE plot showing the distribution of cell clusters.
3. T-SNE visualization of the expression level of main lineages markers. The color key from gray to red indicates low to high gene expression.
4. T-SNE visualization of the expression level of the top markers from each of the cell lineages. The color key from gray to purple indicates low to high gene expression.
5. Dot plot representing the expression level (purple jet) and the number of expressing cells (dot size) of the top markers from each of the cell lineages.


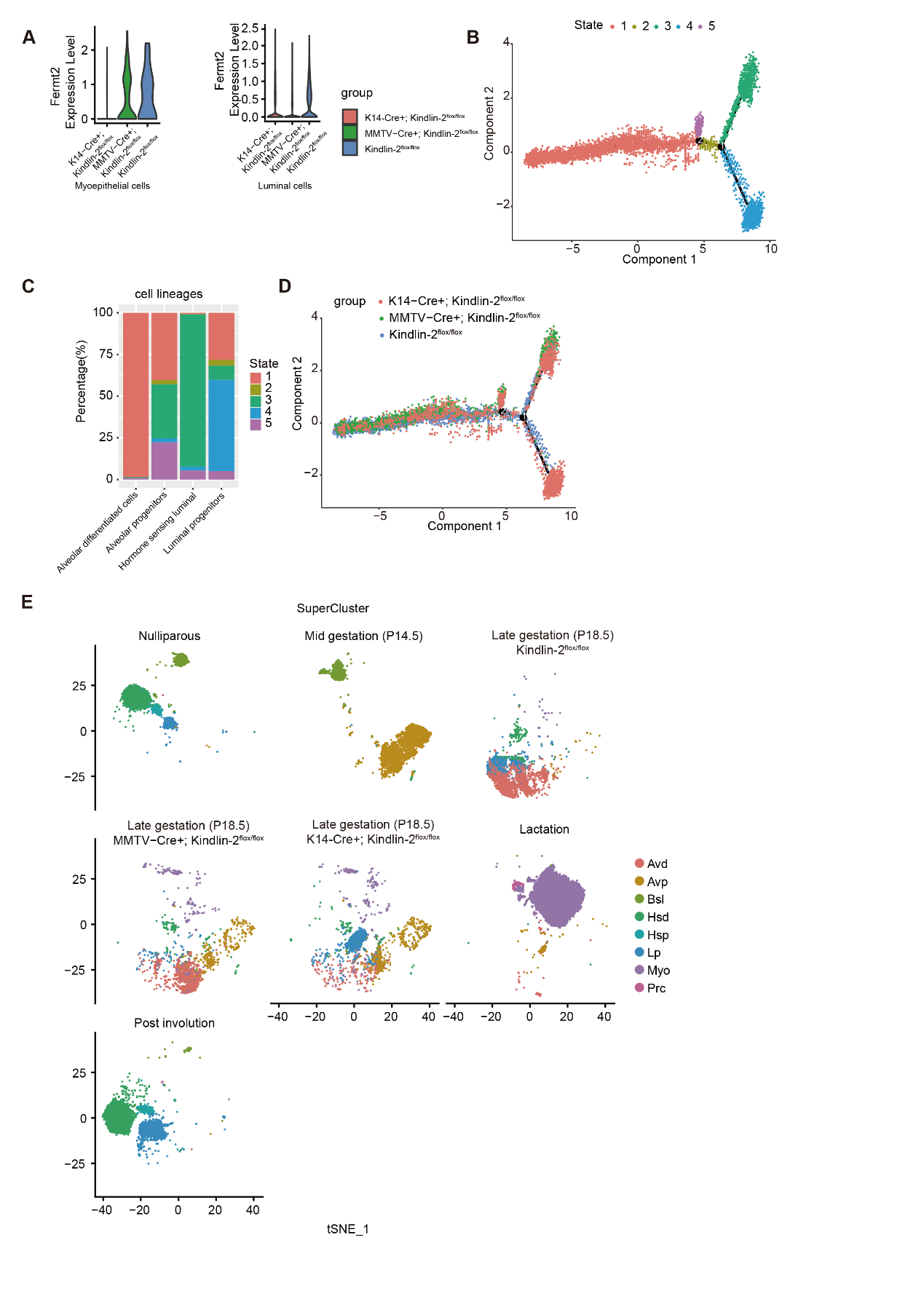


Fig S3. Mammary epithelial cells diversity among Kindlin-2^flox/flox^, K14-Cre+; Kindlin-2^flox/flox^ and MMTV-Cre+; Kindlin-2^flox/flox^ pregnant mouse mammary glands.

A) Violin plots showing *FERMT2* expression levels in mammary epithelial cells from each genotype.

B) Developmental trajectory of luminal cell lineages grouped by states.

C) Bar plot showing the distribution of states among the cell lineages.

D) Developmental trajectory of luminal cell lineages grouped by genotypes.

E) T-SNE plot showing the distribution of the cell lineages within the integrated dataset, split by time points and genotypes.


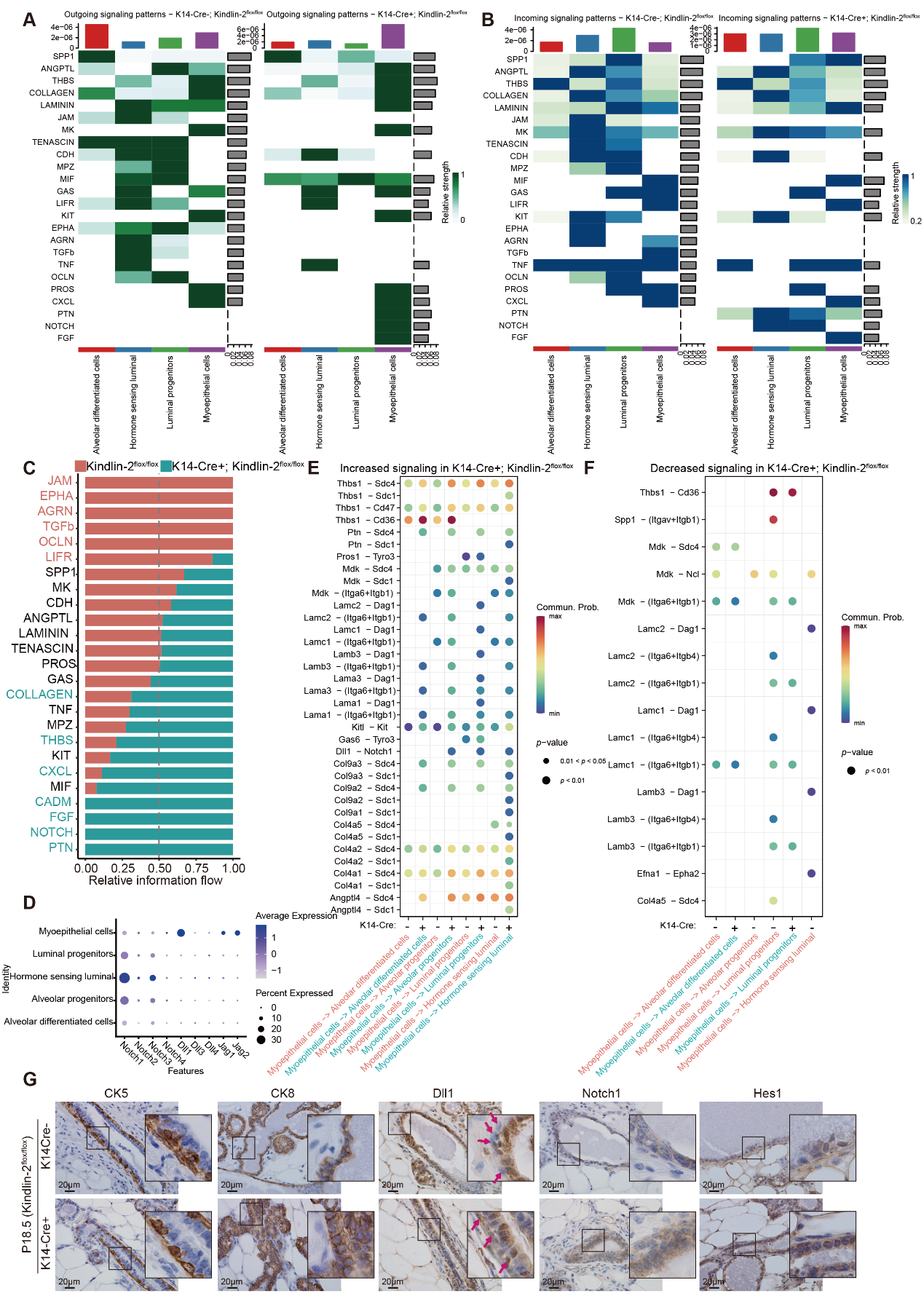


Fig S4. Cell interactions differences of mammary epithelial cells between K14-Cre+; Kindlin-2^flox/flox^ and K14-Cre-; Kindlin-2^flox/flox^ pregnant mouse mammary glands.

A, B) Heatmap showing the outgoing and incoming signaling patterns of K14-Cre+; Kindlin-2^flox/flox^ and K14-Cre-; Kindlin-2^flox/flox^ genotype mouse mammary epithelial cells.

C) Bar plot showing significant signaling pathways ranked based on differences within the inferred networks between K14-Cre+; Kindlin-2^flox/flox^ and K14-Cre-; Kindlin-2^flox/flox^ genotype. Red enriched in the K14-Cre-; Kindlin-2^flox/flox^ genotype. Green enriched in the K14-Cre+; Kindlin-2^flox/flox^ genotype.

D) Dot plot representing the expression level (purple jet) and the number of expressing cells (dot size) of Notch pathway canonical ligands and receptors in epithelium.

E) Dot plot showing the significantly increased signaling in K14-Cre+; Kindlin-2^flox/flox^ mouse mammary epithelial cells compared with K14-Cre-; Kindlin-2^flox/flox^ genotype. The color key from blue to red represent the higher communication strength. Dot size represent the significance.

F) Dot plot showing the significantly decreased signaling in K14-Cre+; Kindlin-2^flox/flox^ mouse mammary epithelial cells compared with K14-Cre-; Kindlin-2^flox/flox^ genotype. The color key from blue to red represent the higher communication strength. Dot size represent the significance.

G) Immunohistochemical staining for CK5, CK8, Dll1, Notch1 and Hes1 expression of P18.5 mammary gland in K14-Cre+; Kindlin-2^flox/flox^ and K14-Cre-; Kindlin-2^flox/flox^ females. Scale bar, 20 µm.


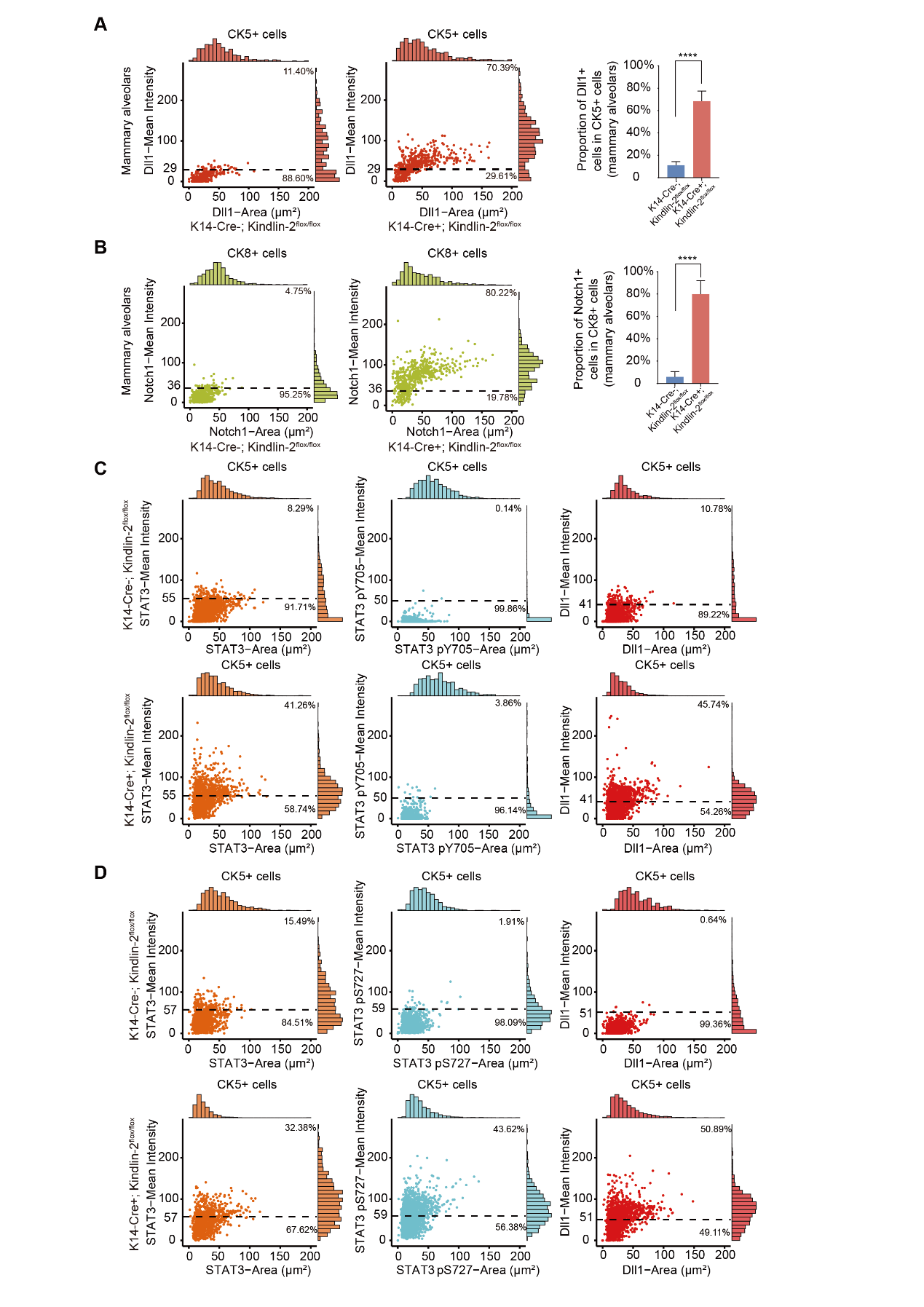


Fig S5. Quantitative statistical analysis of multi-color staining.

A) The scatter plot showing the number of Dll1+ cells in CK5+ cells of P18.5 mammary alveolars in K14-Cre+; Kindlin-2^flox/flox^ and K14-Cre-; Kindlin-2^flox/flox^ females. Statistical analysis showing the proportion of Dll1+ cells in CK5+ cells at P18.5 in K14-Cre+; Kindlin-2^flox/flox^ group compared with K14-Cre-; Kindlin-2^flox/flox^ group (*n*=5 mammary alveolars of view at random per genotype). Statistical testing was performed by unpaired *t*-test. Data are presented as mean values+/− SD. **P* < 0.05, ***P* < 0.01, ****P* < 0.001, *****P* < 0.0001.

B) The scatter plot showing the number of Notch1+ cells in CK8+ cells of P18.5 mammary alveolars in K14-Cre+; Kindlin-2^flox/flox^ and K14-Cre-; Kindlin-2^flox/flox^ females. Statistical analysis showing the proportion of Notch1+ cells in CK8+ cells at P18.5 in K14-Cre+; Kindlin-2^flox/flox^ group compared with K14-Cre-; Kindlin-2^flox/flox^ group (*n*=5 mammary alveolars of view at random per genotype). Statistical testing was performed by unpaired *t*-test. Data are presented as mean values+/− SD. **P* < 0.05, ***P* < 0.01, ****P* < 0.001, *****P* < 0.0001.

C) The scatter plot showing the number of STAT3+ cells in CK5+ cells, STAT3 pY705+ cells in CK5+ cells, and Dll1+ cells in CK5+ cells of P18.5 mammary gland in K14-Cre+; Kindlin-2^flox/flox^ and K14-Cre-; Kindlin-2^flox/flox^ females.

D) The scatter plot showing the number of STAT3+ cells in CK5+ cells, STAT3 pS727+ cells in CK5+ cells, and Dll1+ cells in CK5+ cells of P18.5 mammary gland in K14-Cre+; Kindlin-2^flox/flox^ and K14-Cre-; Kindlin-2^flox/flox^ females.


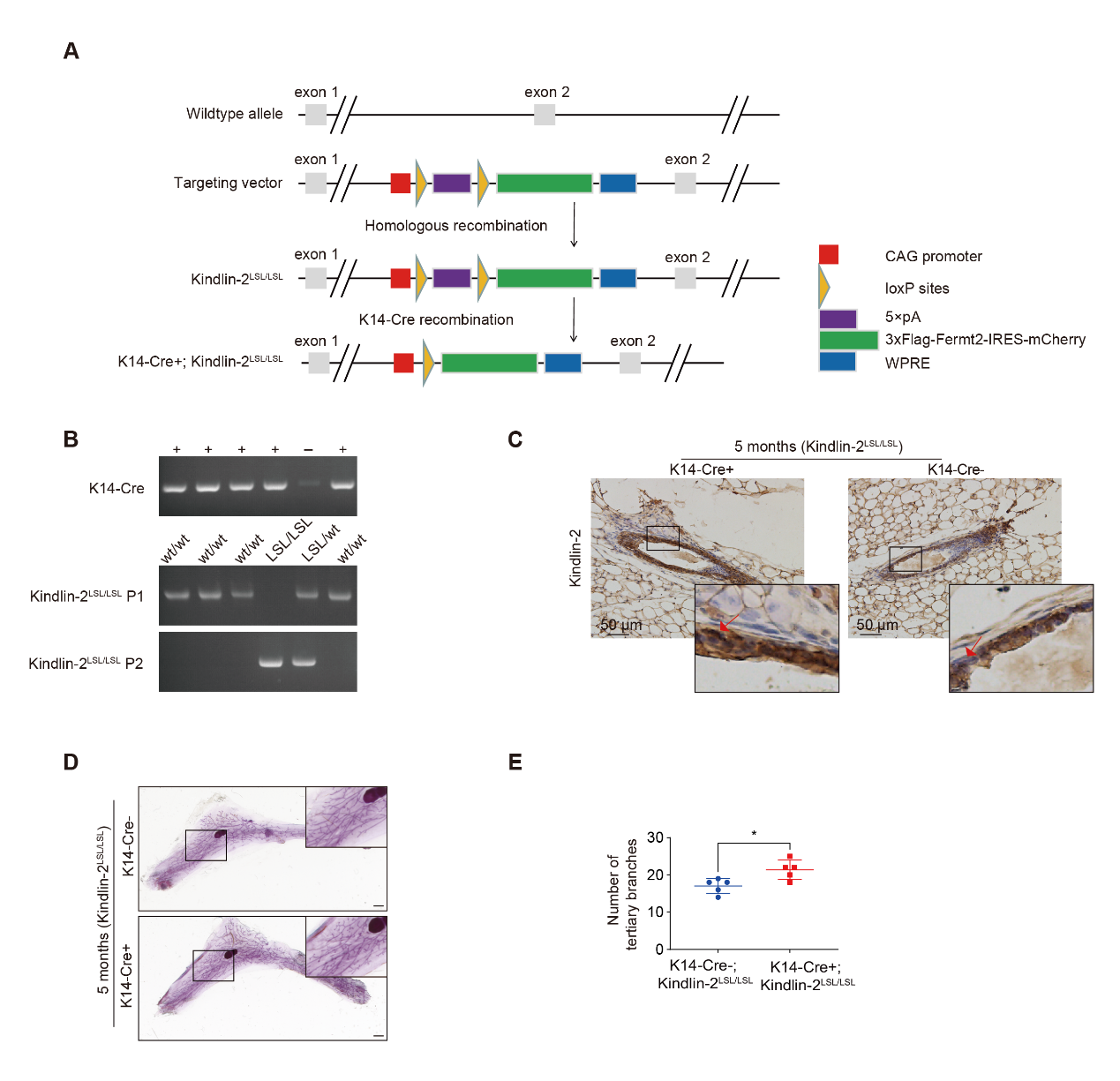


Fig S6. Overexpression of Kindlin-2 promoted mammary gland development in mice.

A) Schematic diagram of the construction of the mice models.

B) Characterizing the genotype by polymerase chain reaction in K14-Cre+; Kindlin-2^LSL/LSL^ or K14-Cre-; Kindlin-2^LSL/LSL^ littermate control mice.­

C) Immunohistochemical staining for Kindlin-2 expression in 5-month-old mammary gland of K14-Cre+; Kindlin-2^LSL/LSL^ and K14-Cre-; Kindlin-2^LSL/LSL^ females. Scale bar, 50 µm.

D) Whole-mounted staining in 5-month-old mammary gland of K14-Cre+; Kindlin-2^LSL/LSL^ or K14-Cre-; Kindlin-2^LSL/LSL^ littermate control mice.

E) Statistical analysis showing the number of tertiary branches in K14 Cre+; Kindlin-2^LSL/LSL^ group compared with K14 Cre-; Kindlin-2^LSL/LSL^ group (*n*=5 fields of view at random per genotype). Statistical testing was performed by unpaired *t*-test. Data are presented as mean values+/− SD. **P* < 0.05.


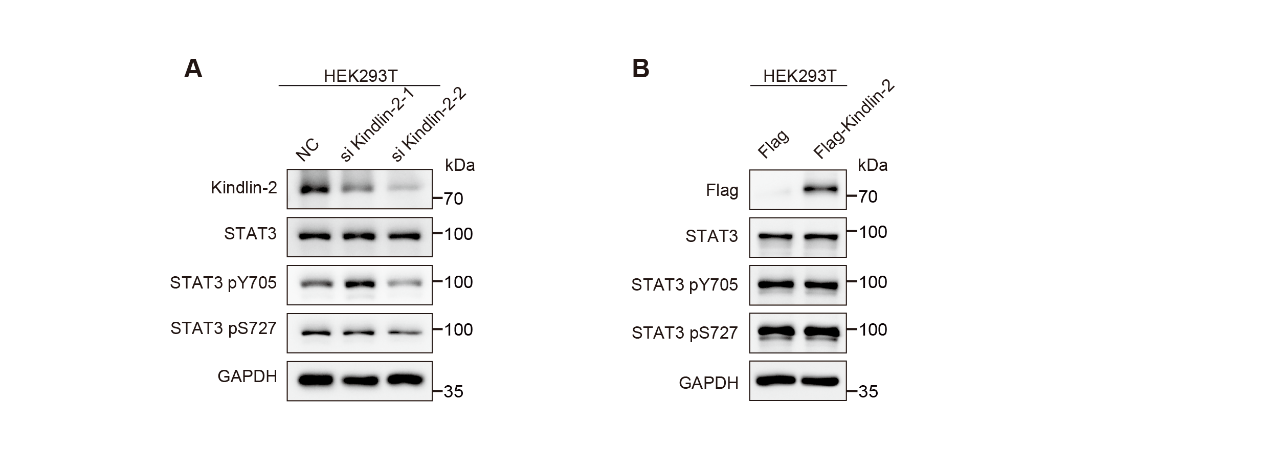
Fig S7. Kindlin-2 specifically regulates phosphorylation of STAT3.

A) Western blot analysed of STAT3, STAT3 pY705, and STAT3 pS727 using specific antibodies through loss of function of Kindlin-2 in the cell lines: HEK293T. GAPDH acted as an internal reference.

B) Western blot analysed of Flag, STAT3, STAT3 pY705, and STAT3 pS727 using specific antibodies through gain of function of Kindlin-2 in the cell lines: HEK293T. GAPDH acted as an internal reference.
