# Supplementary material for: Kindlin-2 in myoepithelium controls luminal progenitor commitment to alveoli in mouse mammary gland
Source: Cell Death Dis. 2023 Oct 13;14(10):675. doi: 10.1038/s41419-023-06184-2 (PMC10576046; doi:10.1038/s41419-023-06184-2)
Supplement: Supplementary file 2 — Original western blots [file 41419_2023_6184_MOESM2_ESM.pdf]

Kindlin-2 in myoepithelium controls luminal progenitor commitment to alveoli in mouse mammary gland

Zhenbin Wang[1#], Lei Zhang[1#], Bing Li[1, 2#], Jiagui Song[1#], Miao Yu[1], Jing Zhang[1], Ceshi Chen[3, 4*], Jun Zhan[1*], Hongquan Zhang[1*]

Supplementary Original Western Blots

Figure 5

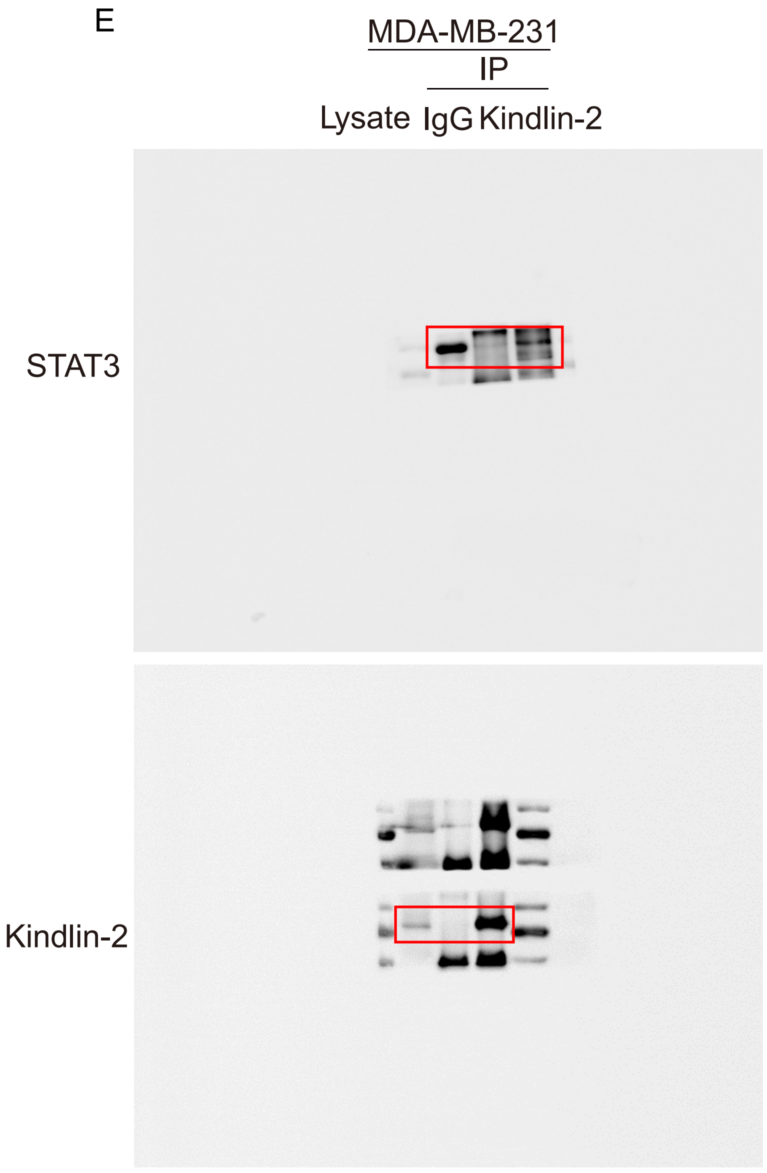

Figure 5

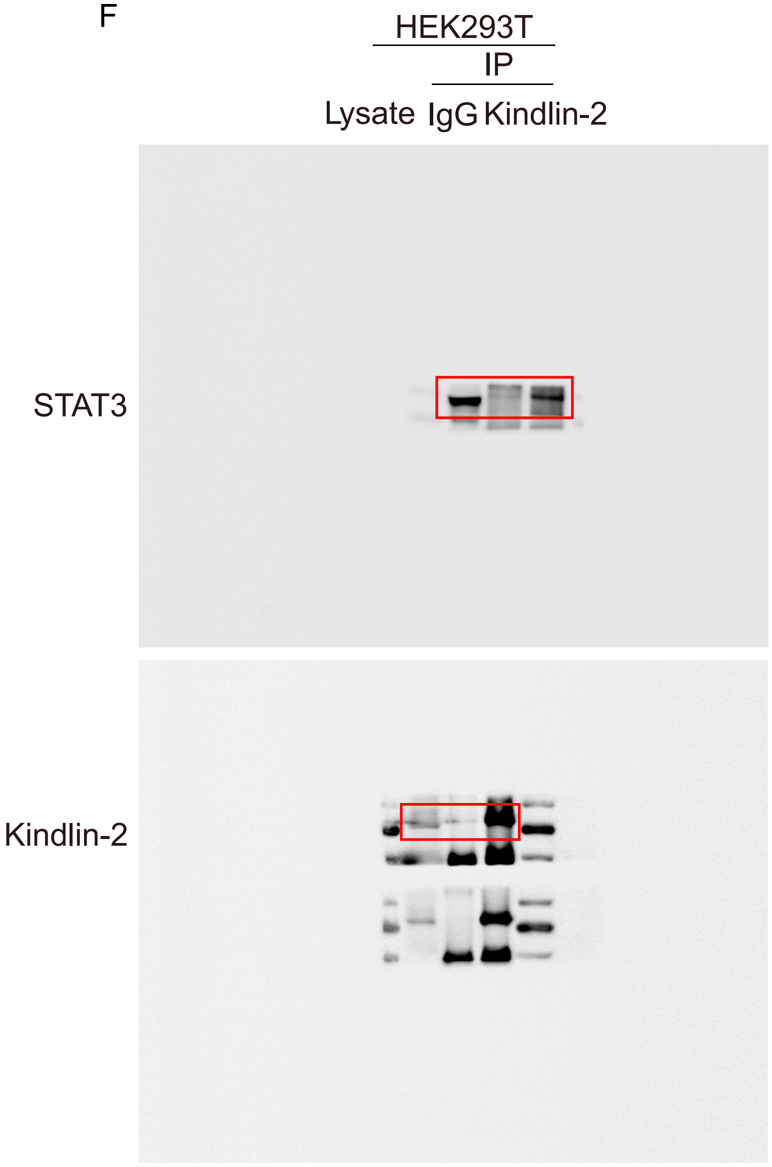

Figure 5

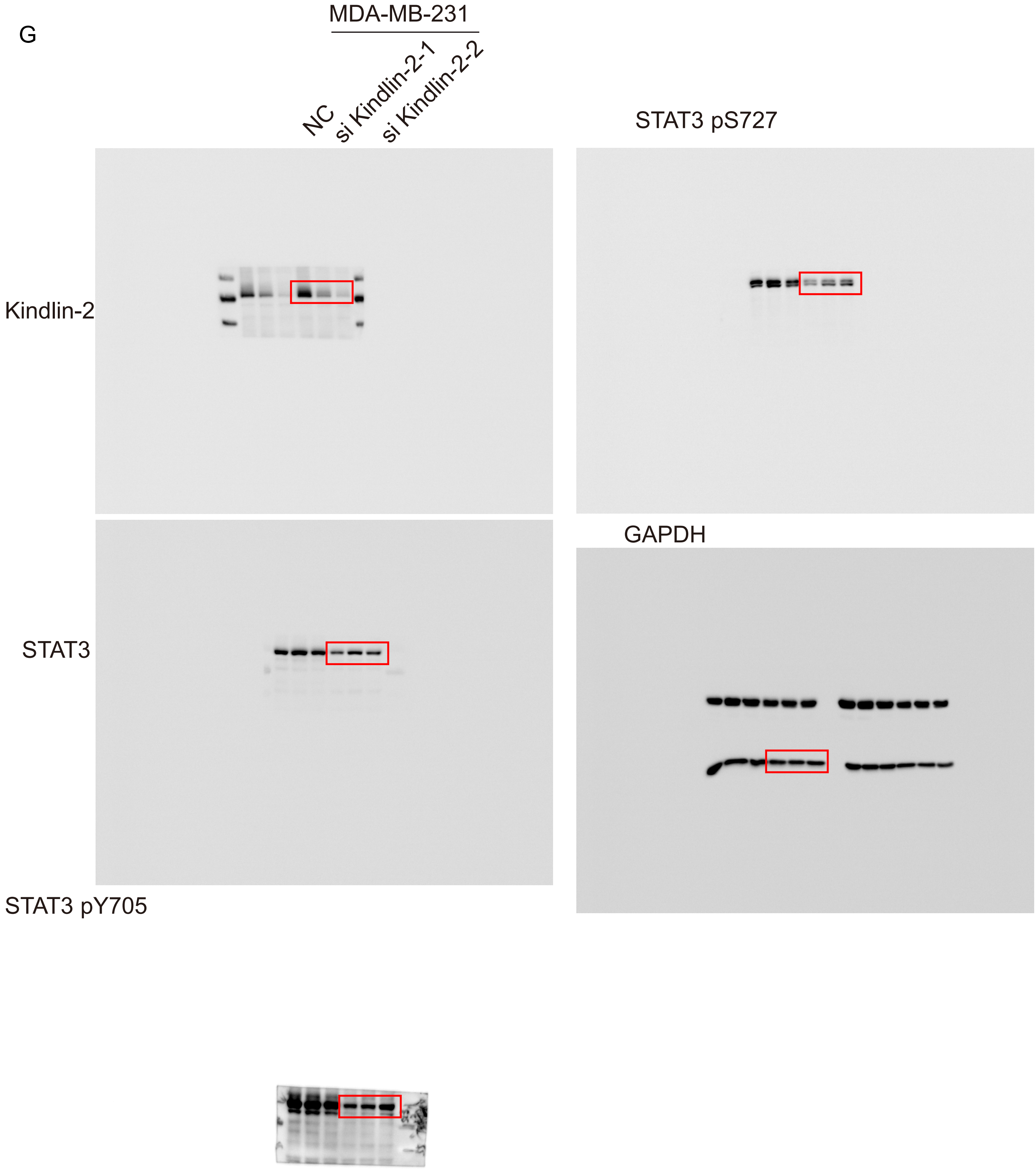
G
MDA-MB-231
NC
si Kindlin-2-1
si Kindlin-2-2
Kindlin-2
STAT3 pS727
STAT3
GAPDH
STAT3 pY705

Figure 5

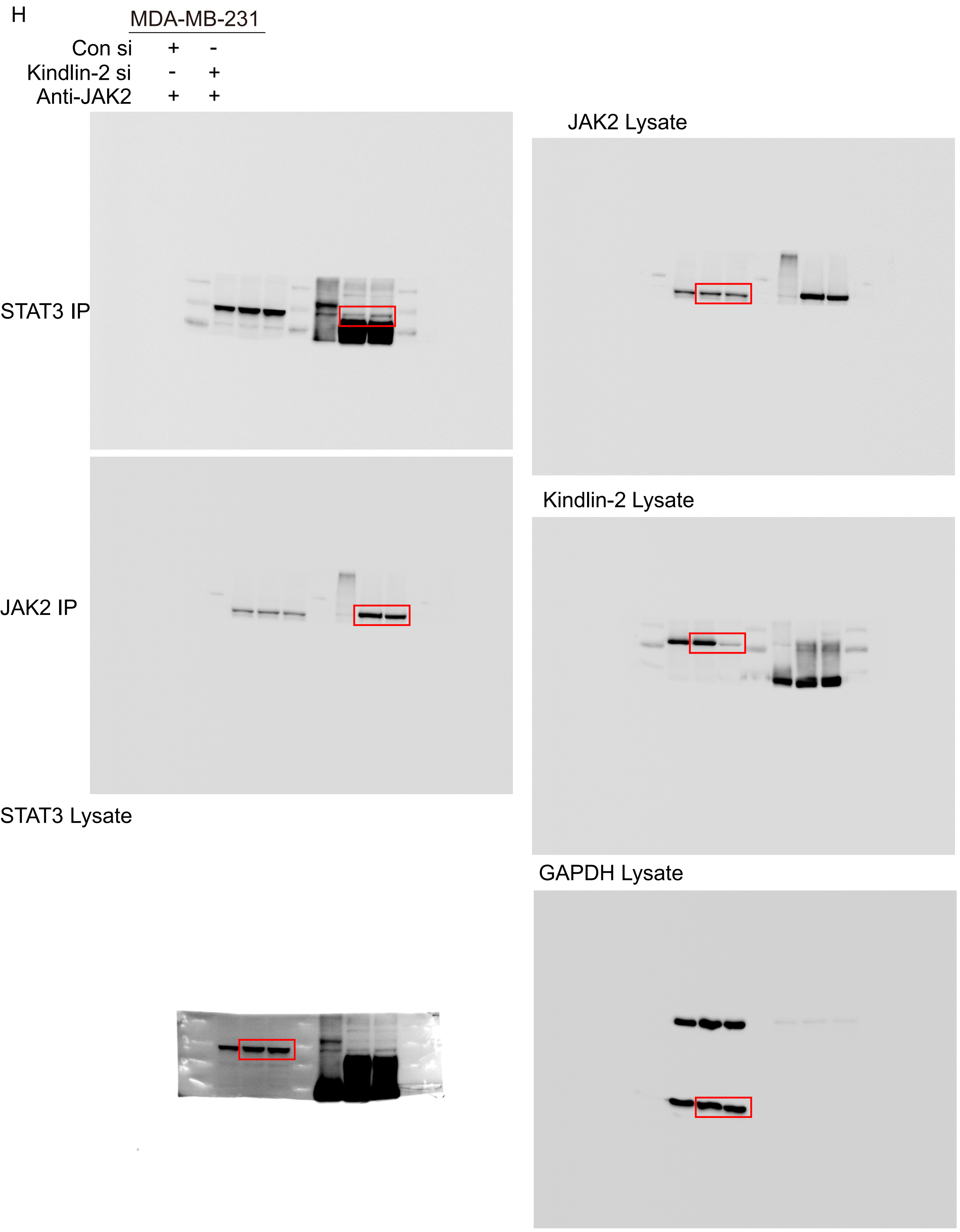
H
MDA-MB-231
Con si + -
Kindlin-2 si - +
Anti-JAK2 + +
STAT3 IP
JAK2 IP
STAT3 Lysate
JAK2 Lysate
Kindlin-2 Lysate
GAPDH Lysate

Figure 5

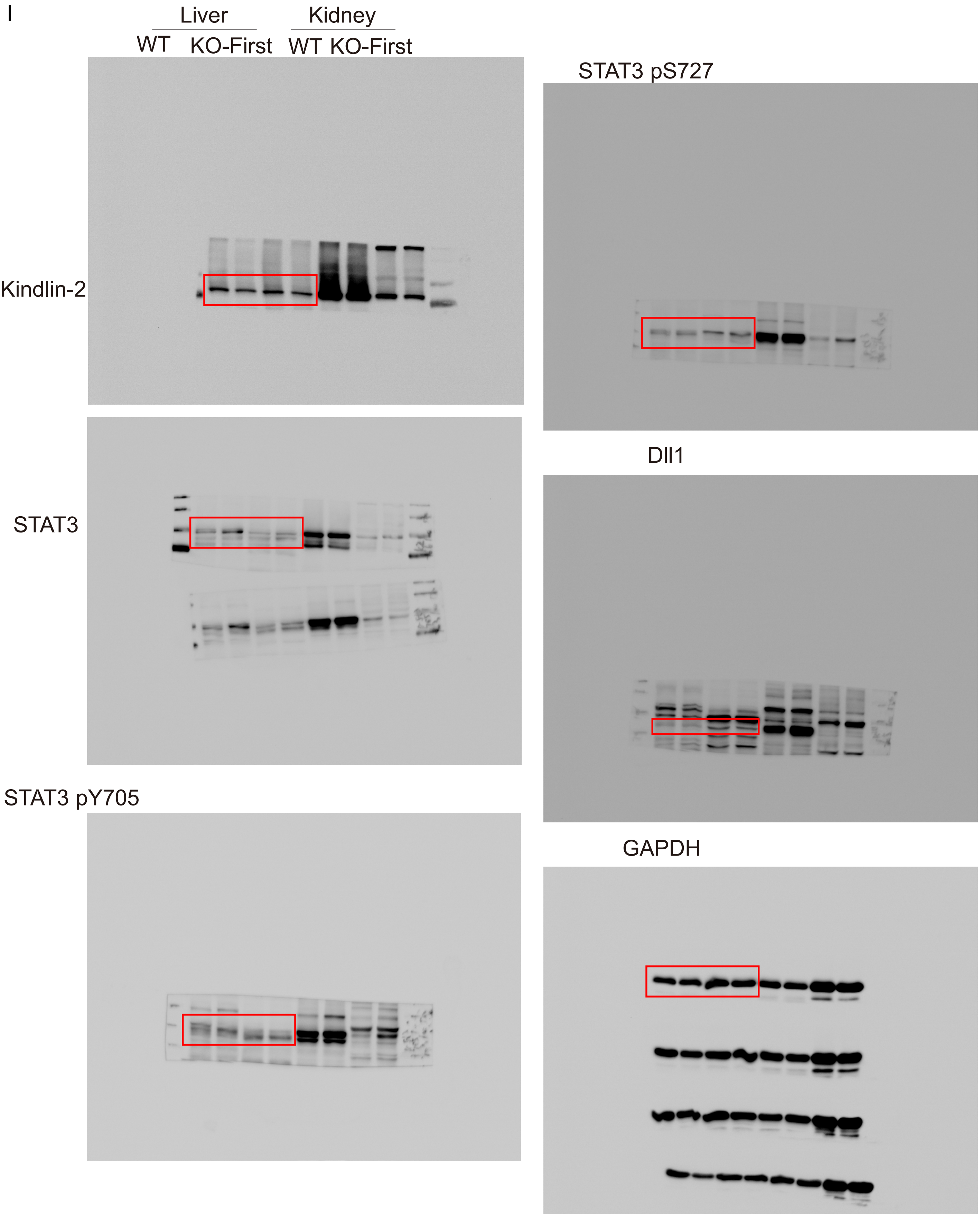
I
Liver
Kidney
WT
KO-First
WT
KO-First
Kindlin-2
STAT3
STAT3 pY705
STAT3 pS727
Dll1
GAPDH

Figure 5

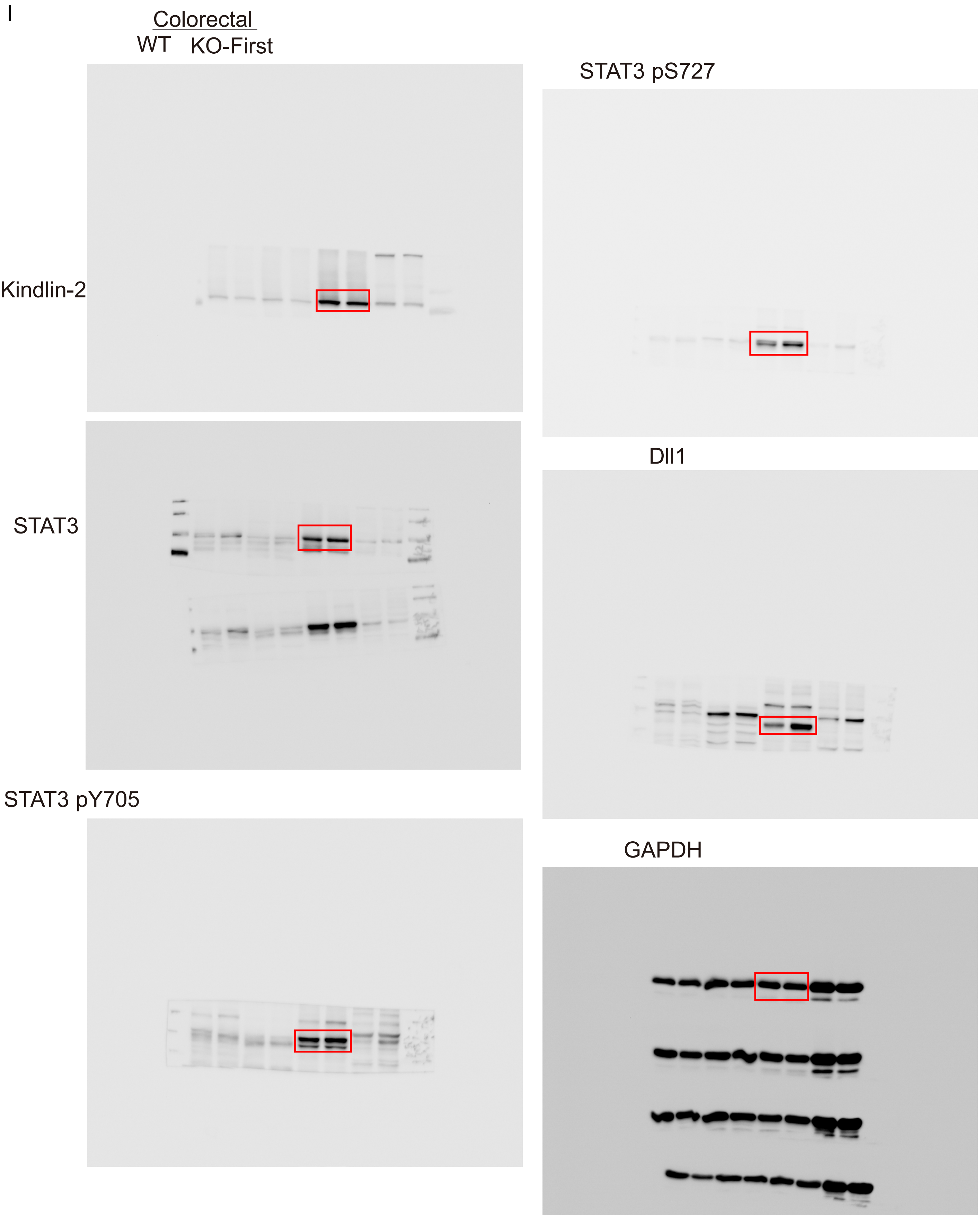
I
Colorectal
WT
KO-First
Kindlin-2
STAT3
STAT3 pY705
STAT3 pS727
Dll1
GAPDH

Figure 5

I

Skeletal Muscle

WT KO-First

Kindlin-2

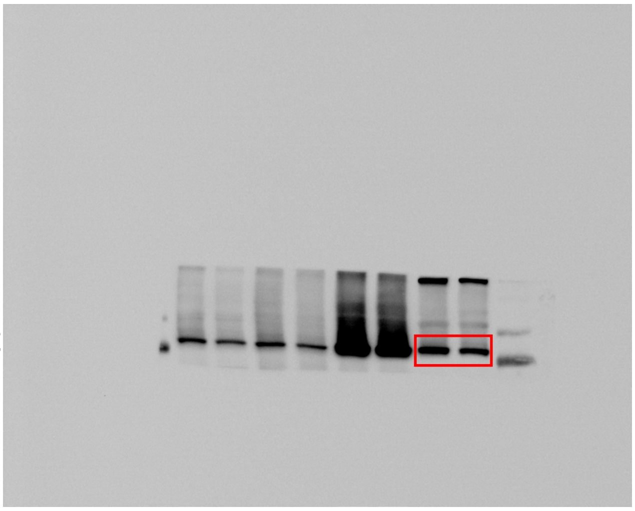

STAT3

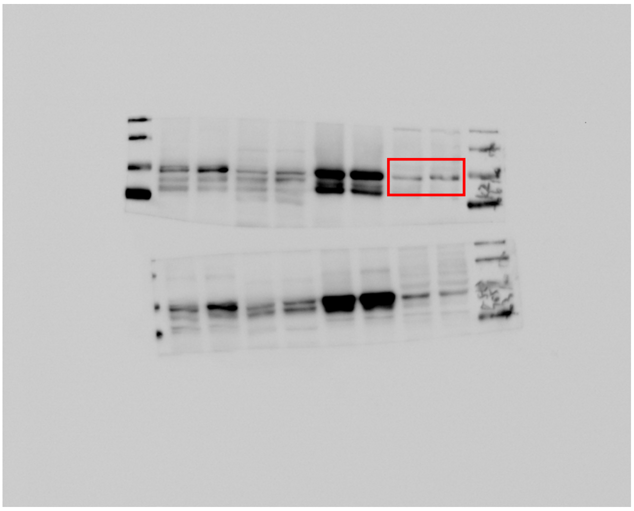

STAT3 pY705

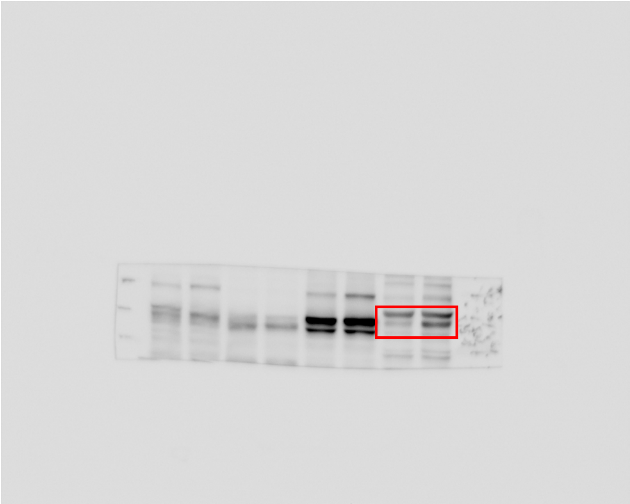

STAT3 pS727

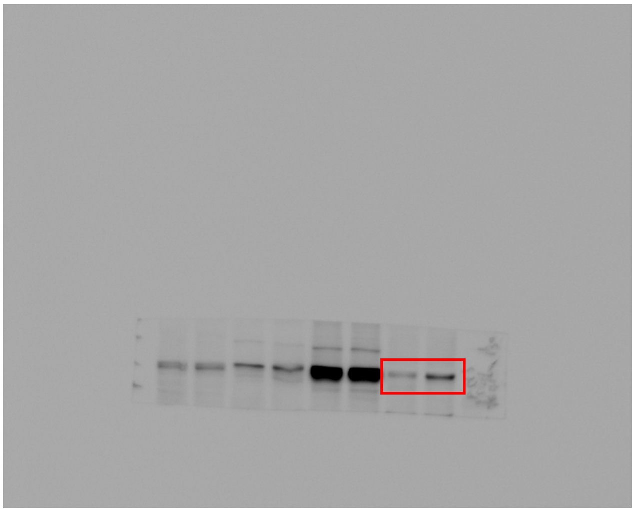

Dll1

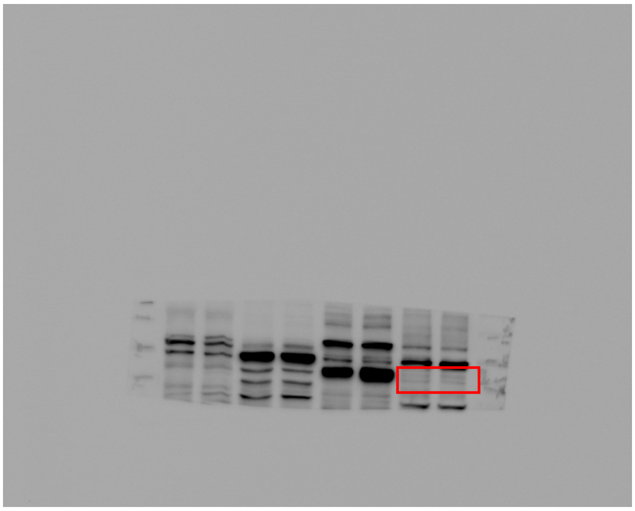

GAPDH

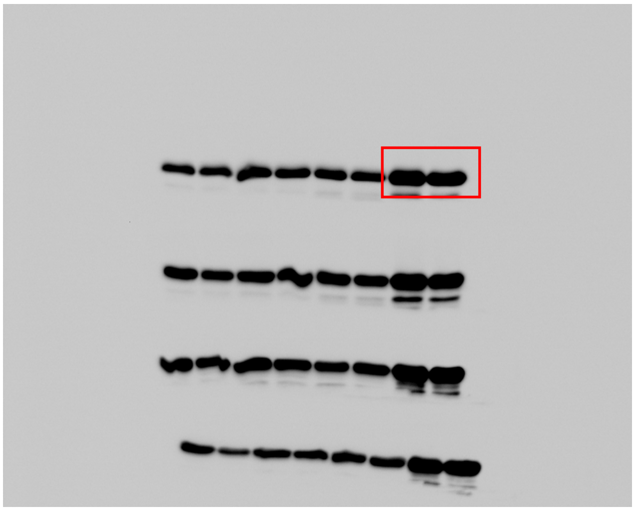

Figure S1

C

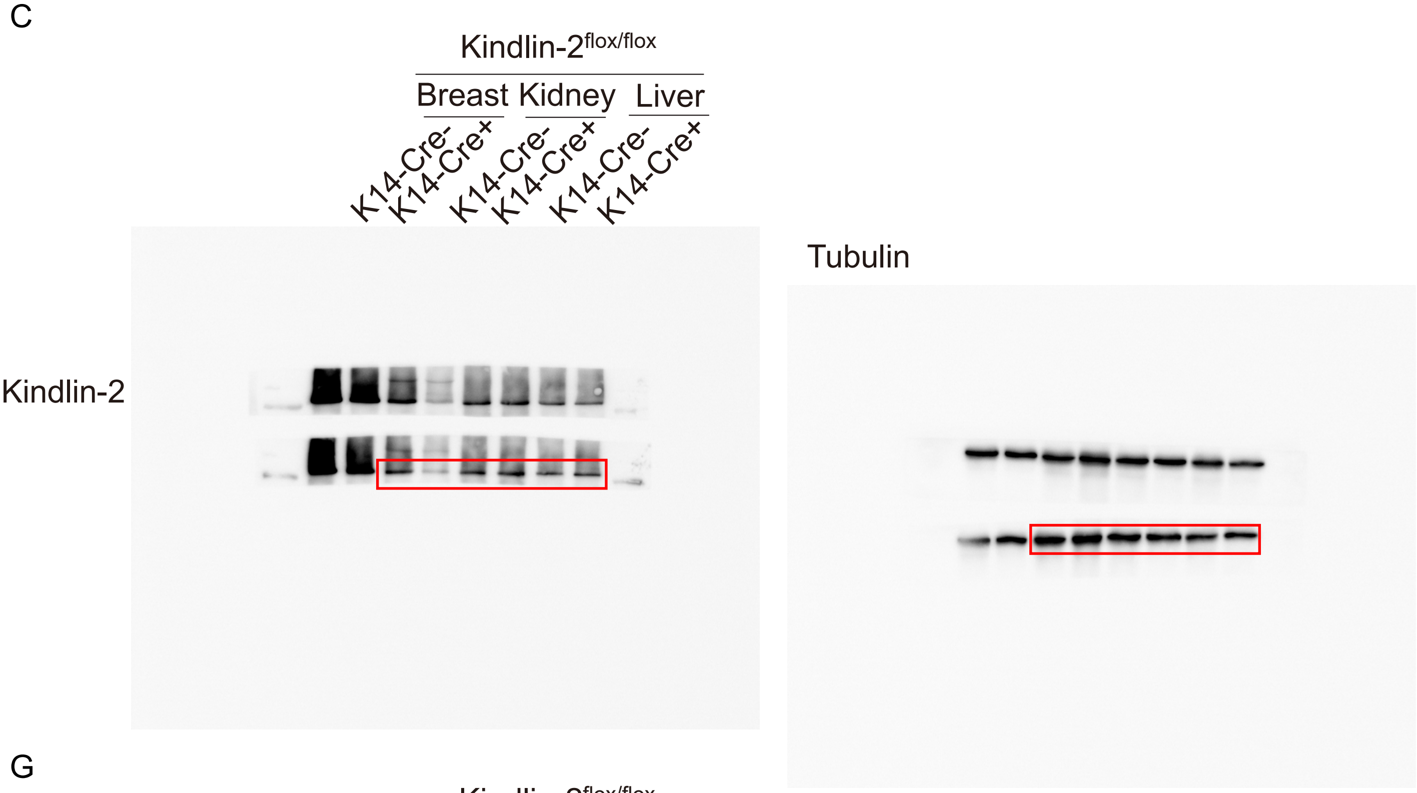

Kindlin-2flox/flox
Breast Kidney Liver
K14-Cre-
K14-Cre+
K14-Cre-
K14-Cre+
K14-Cre-
K14-Cre+
Kindlin-2
Tubulin

G

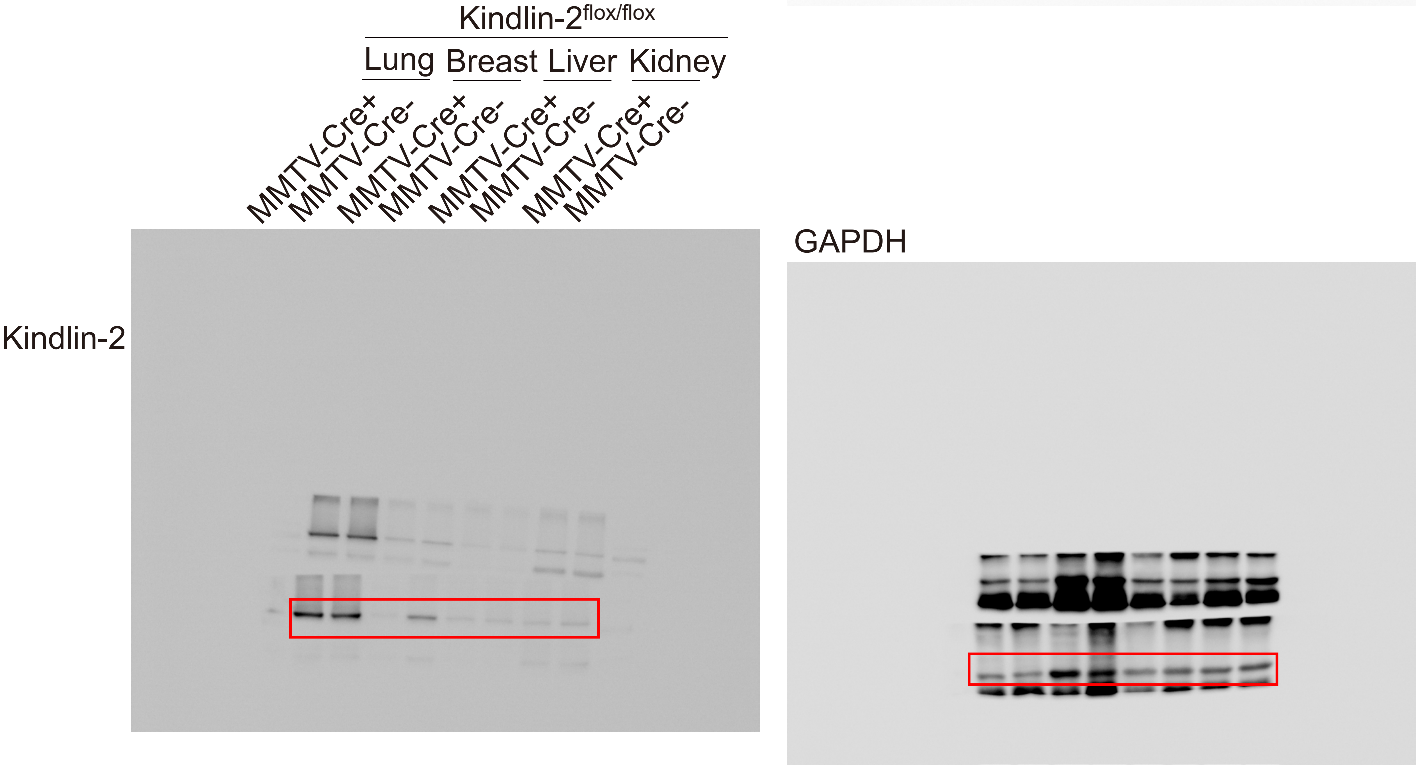

Kindlin-2flox/flox
Lung Breast Liver Kidney
MMTV-Cre+
MMTV-Cre-
MMTV-Cre+
MMTV-Cre-
MMTV-Cre+
MMTV-Cre-
MMTV-Cre+
MMTV-Cre-
Kindlin-2
GAPDH

Figure S7

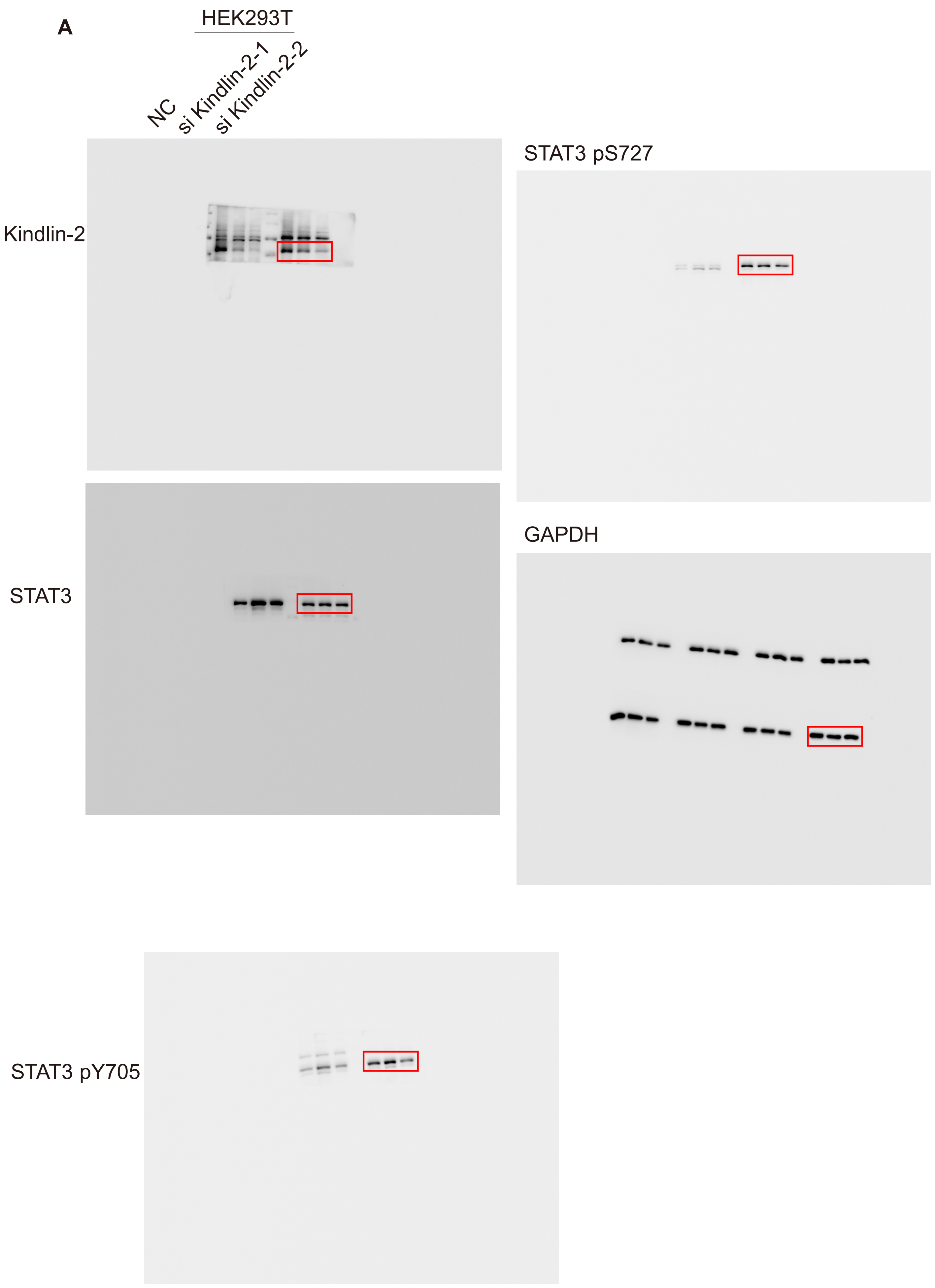
A
HEK293T
NC
si Kindlin-2-1
si Kindlin-2-2
Kindlin-2
STAT3 pS727
STAT3
GAPDH
STAT3 pY705

Figure S7

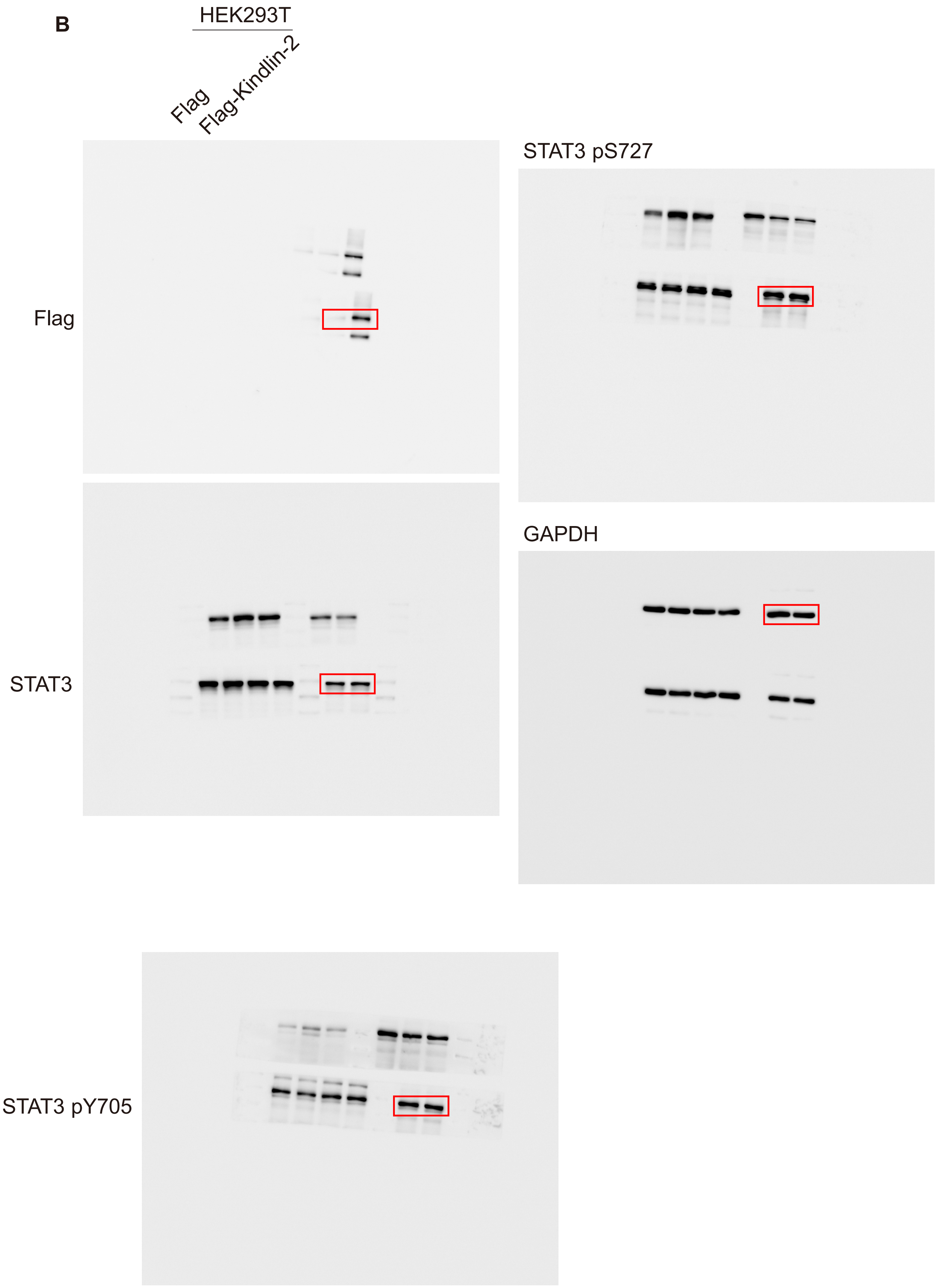
B
HEK293T
Flag
Flag-Kindlin-2
Flag
STAT3 pS727
STAT3
GAPDH
STAT3 pY705
